# *Legionella pelazae* sp. nov. and *Legionella navarrensis* sp. nov., two novel *Legionella* species, reveal deep genomic diversification within engineered water systems

**DOI:** 10.3389/fmicb.2026.1828313

**Published:** 2026-09-09

**Authors:** Juana María González-Rubio, Víctor Manuel López Molina, Silvia Cianca Peña, Almudena Cascajero, Sara Monzón, Isabel Cuesta, Fernando González-Camacho

**Affiliations:** 1 Legionella Reference Laboratory, National Centre for Microbiology, Instituto de Salud Carlos III, Majadahonda, Madrid, Spain; 2 Bioinformatics Unit, Scientific Core Facilities, Instituto de Salud Carlos III, Majadahonda, Madrid, Spain; 3 Microbiology Unit, Public Health Laboratory, Institute of Public Health and Occupational Health of Navarra (ISPLN), Navarra, Spain

**Keywords:** cellular fatty acid, environmental microbiology, *Legionella* non-*pneumophila*, *mip* gene, phylogenomics, whole genome sequencing

## Abstract

Members of the genus *Legionella* are Gram-negative aquatic bacteria widely distributed in natural and engineered aquatic environments, yet the genomic diversity of environmental representatives remains incompletely resolved. During routine surveillance of two independent engineered water systems in Spain, two strains were recovered that could not be assigned to any described species using conventional molecular markers. To determine their taxonomic position, we applied a polyphasic approach integrating 16S rRNA and *mip* gene sequencing, whole-genome sequencing, phylogenomic reconstruction based on conserved core proteins, genome-based relatedness indices, and phenotypic and chemotaxonomic characterization. Phylogenetic analyses based on 16S rRNA and *mip* gene sequences placed both isolates within the genus *Legionella* while clearly separating them from recognized taxa. Genome-scale analyses confirmed deep divergence, with average nucleotide identity values of 85.01 % for *Legionella pelazae* sp. nov. and 70.37 % for *Legionella navarrensis* sp. nov. relative to their closest described species. The strains exhibited distinct genome architectures, including G+C contents of 35.52 and 48.72 mol%, and showed reproducible physiological and membrane lipid differences consistent with ecological differentiation. No acquired antimicrobial resistance determinants were detected. Taken together, these results reveal substantial intrageneric diversification among environmental *Legionella* populations inhabiting engineered water systems. On the basis of combined genomic, phylogenetic and phenotypic evidence, the strains represent two novel species within the genus, for which the names *Legionella pelazae* sp. nov. (CNM-1927-20^T^ = CECT 31282=CCUG 78397^T^) and *Legionella navarrensis* sp. nov. (CNM-4043-24^T^ = CECT 31283 = CCUG 78398^T^) are proposed.

## 1 Introduction

Members of the genus *Legionella* are ubiquitous Gram-negative bacteria widely distributed in freshwater ecosystems and engineered water systems (EWS; Fields et al., 2002). Within natural environments, they persist as intracellular parasites of free-living amoebae and other protozoa, a lifestyle that has shaped the evolution of complex secretion systems and stress adaptation mechanisms (Gomez-Valero and Buchrieser, 2019; Price et al., 2024; Richards et al., 2013). In EWS, including hot water distribution networks, cooling towers, spas and healthcare facilities, *Legionella* species may proliferate under favorable physicochemical conditions, particularly in the presence of biofilms, stagnation, and elevated temperatures (Falkinham, 2020; Yao et al., 2024).

EWS represent highly dynamic ecological niches shaped by repeated thermal fluctuations, chemical disinfection regimes, hydraulic disturbances and nutrient limitation (Nisar et al., 2020). These selective pressures can influence microbial community structure and may promote genomic diversification and physiological specialization (Newton et al., 2018). Membrane composition, oxidative stress responses, secretion systems and metabolic flexibility are all traits that may contribute to persistence in such environments (Oliva et al., 2018). Consequently, EWS may act not only as reservoirs but also as selective landscapes fostering diversification within environmental *Legionella* populations (Yao et al., 2024).

Although *Legionella pneumophila* remains the primary etiological agent of legionellosis, an increasing number of non-*pneumophila* species have been detected in environmental surveillance programs, and several have been associated with sporadic human infections (Hammes et al., 2025). Nevertheless, most environmental *Legionella* diversity remains poorly characterized, and the ecological and evolutionary dynamics of non-clinical lineages are incompletely understood. Traditional identification methods based on partial gene sequencing, such as 16S rRNA or macrophage infectivity potentiator (*mip*, Ratcliff et al., 1998), often lack sufficient resolution to capture deep genomic divergence, potentially underestimating species-level diversity within the genus (Chun and Rainey, 2014; Kim et al., 2014; Richter and Rosselló-Móra, 2009).

The widespread implementation of whole-genome sequencing has transformed bacterial systematics by enabling genome-wide comparisons that more accurately resolve evolutionary relationships. Genome-based metrics such as average nucleotide identity (ANI) and digital DNA–DNA hybridization (dDDH), combined with phylogenomic reconstruction, have refined species boundaries and revealed substantial heterogeneity within established genera (Chun and Rainey, 2014; Meier-Kolthoff et al., 2013; Richter and Rosselló-Móra, 2009). In *Legionella*, comparative genomics has demonstrated the presence of conserved intracellular survival mechanisms alongside extensive accessory genome variation, suggesting ongoing evolutionary diversification driven by environmental adaptation and horizontal gene exchange (Gomez-Valero et al., 2011).

During routine environmental surveillance of two independent EWS in Spain, two isolates were recovered that could not be confidently assigned to any described *Legionella* species based on conventional molecular markers. In this study, we applied a comprehensive polyphasic approach integrating whole-genome sequencing, phylogenomics, genome-based relatedness indices, phenotypic characterization and chemotaxonomic profiling to resolve their taxonomic position. Our findings reveal substantial genomic divergence within the genus and highlight ecological differentiation among environmental strains inhabiting EWS. On the basis of combined genomic and phenotypic evidence, these strains represent two previously unrecognized species within *Legionella*, thereby expanding the known diversity and evolutionary breadth of the genus.

## 2 Materials and methods

### 2.1 Sampling, isolation and cultivation

Water sampling and subsequent isolation procedures were carried out in compliance with Spanish Royal Decree 487/2022 (RD 487/2022), UNE 100030:2017 and UNE-EN ISO 11731:2017 standards, which regulate the prevention and control of *Legionella* and its detection and enumeration. Briefly, 1 L of water sample was filtered through a polycarbonate filter (0.2 μm pore size and 47 mm diameter, Millipore^®^, Millipore Sigma, Burlington, MA, USA) and the filter was then eluted in 10 mL of 1/40 Ringer's solution. An aliquot of 0.1-0.5 mL of the concentrate was subjected to heat shock (50 C/ 30 min) and acid-treatment (Buffer KCl/HCl 2 M, 5 min). Treated and untreated samples were cultured on *Legionella* Buffered Charcoal Yeast Extract (BCYE) agar supplemented with glycine, vancomycin, polymyxin B and cycloheximide (GVPC; Thermo Fisher Scientific, Diagnostic, Ltd., Basingstoke, UK) and incubated at 37 C for 7–10 days. Colonies with morphology compatible with *Legionella* were subcultured onto BCYE agar supplemented with L-cysteine (BCYEα) and BCYE agar without L-cysteine (BCYEα-cys) to verify the L-cysteine growth requirement. Isolates showing growth on BCYEα but not on BCYEα-cys were subjected to latex immunoagglutination (OXOID, Oxoid Ltd., Basingstoke, UK).

Strain CNM-1927-20^T^ was isolated prior to heat treatment on 2 March 2020 from a spa located at 42°3′35”N 1°4′25”W, in a municipality of the Comunidad Foral de Navarra, Spain, as part of routine control and surveillance program. The strain was recovered from a tap supplying cooled thermal water in the spa bath gallery. The water temperature at the time of sampling was 45.5 C; and hydrogen peroxide (H_2_O_2_) was used as the biocide at the facility, with a residual concentration of 20 ppm. The isolate was recovered prior to heat treatment during laboratory processing, indicating its presence within the active microbial community of the system. Strain CNM-4043-24^T^ was isolated on 14 February 2024 at a rural tourism accommodation located at 42°5′03”N 2°2′20”W in the Comunidad Foral de Navarra, Spain, during routine control and surveillance sampling. The strain originated from a sample collected from the hot water return line with a water temperature of 49.2 C at the time of sampling. Sodium hypochlorite was used as the biocide at this facility, resulting in a residual chlorine concentration of 0.49 ppm. Positives isolates were obtained prior to acid treatment.

### 2.2 Sequencing of 16S rRNA and *mip* genes

To obtain a preliminary taxonomic assignment and assess the phylogenetic position of the isolates within the genus *Legionella*, partial sequences of the 16S rRNA (~1,400 bp) and *mip* (~600 bp) genes were analyzed.

Strains CNM-1927-20^T^ and CNM-4043-24^T^ were identified by PCR amplification and sequencing of the *mip* and 16S rRNA genes. Genomic DNA was extracted from pure cultures using the QiAcube automated system (Qiagen, Hilden, Germany). PCR amplification and sequencing of both genes were performed using previously described universal primers (Ratcliff et al., 1998). Sequence identification and percentage identity were determined using the Basic Local Alignment Search Tool (BLAST) from National Center for Biotechnology Information (NCBI).

For phylogenetic analysis, 16S rRNA and *mip* gene sequences from the 68 validly published *Legionella* species available in public NCBI Assembly database (GenBank/RefSeq) were included. Multiple sequence alignments were generated using MUSCLE algorithm (Edgar, 2004). Phylogenetic trees were constructed using the maximum likelihood method based on the Tamura-Nei model of nucleotide substitution (Tamura and Nei, 1993), with bootstrap support values calculated from 1,000 replicates. These phylogenetic analyses were performed using MEGA X (Kumar et al., 2018). To improve branch support estimation, additional phylogenetic analyses were performed using IQ-TREE v2.1.4 (Minh et al., 2020) with 1,000 ultrafast bootstrap (UFBoot) and SH-aLRT replicates. Nodes with support values lower than 70 % were not displayed in the final trees.

### 2.3 Whole genome analyses and phylogeny

Whole-genome sequencing was performed to enable genome-based taxonomic characterization, assess genome quality, and support phylogenomic analysis.

Genomic DNA was extracted from pure cultures of both strains. The extracted DNA was used to prepare short-read genomic libraries, which were sequenced using the Illumina NextSeq 500 System (Illumina, San Diego, CA, USA), generating 150 bp paired-end reads.

Whole-genome sequencing reads were processed using the nf-core/bacass pipeline v2.3.1 (Vm et al., 2024). Quality assessment of raw reads was performed with FastQC v0.12.1, which were then trimmed with fastp v0.23.4 (Chen et al., 2018), applying a minimum Phred quality score of 20. *De novo* assembly was performed with Unicycler v0.5.0 (Wick et al., 2017) and assembly quality metrics were evaluated using QUAST v5.2.0 (Gurevich et al., 2013).

Genome completeness and contamination estimates were obtained using CheckM v1.2.4 (Parks et al., 2015) as implemented in the NCBI Prokaryotic Genome Annotation Pipeline (PGAP) during genome submission. Potential contamination and taxonomic consistency were further evaluated using KmerFinder v3.0.2 (Clausen et al., 2018; Hasman et al., 2014; Larsen et al., 2014). To complement these analyses, taxonomic profiling of raw reads was performed using the nf-core/taxprofiler pipeline (v1.1.8; Stamouli et al., 2023), which classifies reads against the Kraken (Wood et al., 2019), Bracken (Lu et al., 2017), Centrifuge (Kim et al., 2016) and Kaiju databases (Menzel et al., 2016).

The presence of the *mip* gene and the 16S rRNA gene was determined using BLAST v2.16.0 (Camacho et al., 2009).

Genome annotation was conducted with Prokka v1.14.6 (Seemann, 2014) to predict coding sequences and structural features and results were summarized using MultiQC v1.21.0 (Ewels et al., 2016).

Protein-coding sequences were functionally annotated using eggNOG-mapper v2.1.13 (Cantalapiedra et al., 2021) to characterize the functional genomic repertoire against the eggNOG database (version 5.0.2, Huerta-Cepas et al., 2019) to assign orthology relationships and functional annotations. Sequence similarity searches were conducted using DIAMOND v2.0.15 (Buchfink et al., 2021). Functional categories were assigned based on Clusters of Orthologous Groups (COG) classification. For each genome, the distribution of COG categories was calculated by counting the number of proteins assigned to each functional class. Proteins assigned to multiple COG categories were included in all corresponding functional groups. Results were normalized as percentages of total COG assignments to allow comparison between strains. Comparative analysis between strains was performed by visualizing the relative abundance of COG functional categories using bar plots generated in Python v3.9 (Python Software Foundation) employing the pandas v2.3.3 (McKinner, 2010) and matplotlib v3.9.1 (Hunter, 2007) libraries.

### 2.4 Antimicrobial resistance, virulence profiling, plasmid identification and comparative genomics

Screening for antimicrobial resistance (AMR) and virulence-associated genes was carried out to characterize the functional genomic repertoire of the strains and to identify traits potentially relevant to their ecology and biosafety.

The novel strains were screened for AMR and virulence genes using ARIBA v2.14.6 (Hunt et al., 2017). AMR genes were identified using the Comprehensive Antibiotic Resistance Database (CARD; Alcock et al., 2023), and virulence factor were assessed using the Virulence Factor Database (VFDB; Chen et al., 2005). Results from ARIBA were complemented using AMRFinderPlus v3.12.8 (Feldgarden et al., 2021), which detects resistance genes and resistance-associated point mutations based on NCBI's curated Reference Gene Database and a collection of Hidden Markov Models. Default parameters were used for all tools, including the similarity and coverage thresholds implemented internally by each algorithm and database.

To account for potential sequence divergence in the novel strains, additional screening was performed using ABRicate v1.4.0 (Seemann, T., ABRicate, Github, available at https://github.com/tseemann/abricate) against the Virulence Factor Database (VFDB) and the NCBI AMRFinderPlus database. In this analysis, relaxed thresholds of ≥60% nucleotide identity and ≥80% coverage were applied to enable the detection of distant homologs. Hits obtained under these criteria were interpreted as putative homologs rather than confirmed functional determinants. The resulting data were summarized as presence–absence matrices for comparative analysis between the novel strains. Comparative analyses included genomes of closely related *Legionella* species retrieved from NCBI, which were screened under identical parameters.

Comparative genomic analyses were performed to determine the evolutionary relationships of the novel strains with recognized *Legionella* species and to assess their genomic distinctiveness.

For the identification of potential plasmid sequences, PlasmidID v1.6.5 (Monzon et al., 2021, available at https://github.com/BU-ISCIII/plasmidID) was employed, which maps trimmed reads over plasmid database sequences, then clusters the k-mer-filtered and most-covered sequences by identity to avoid redundancy and then uses the longest sequences as a scaffold for plasmid reconstruction. Additionally, ARIBA v2.14.6 (Hunt et al., 2017) was used for this procedure, by means of the PlasmidFinder database (Carattoli et al., 2014).

Phylogenetic relationships between the novel strains and representative *Legionella* genomes were assessed using a core genome multilocus sequence typing (cgMLST) approach, providing a standardized genome-wide comparison based on shared core loci. A cgMLST schema was constructed and evaluated using chewBBACA v3.3.3 (Silva et al., 2018). All publicly available *Legionella* genomes (68 genomes) were initially screened for inclusion in the core-genome analysis. Fourteen genomes were excluded due to a insufficient number of shared loci and poor orthologous gene detection requirements of the chewBBACA schema evaluation, resulting in an inadequate number of loci for reliable incorporation into the core-genome dataset. The genomes retained for phylogenomic reconstruction are listed in Supplementary Data Sheet 1. The final scheme comprised 436 core loci for 54 *Legionella* genomes and was used to determine allelic profiles for the analyzed strains. The translated core-genome alignment generated with by chewBBACA was then used to create an amino acid matrix with pairwise distances using MEGA v11.0.13 (Tamura et al., 2021), to then infer a maximum-likelihood phylogenetic tree using IQTREE (Minh et al., 2020) v2.1.4 under the LG+F+R6 (Le and Gascuel, 2008) amino acid substitution model. Branch support was assessed with 1,000 bootstrap replicates. An additional phylogenomic reconstruction based on conserved bacterial proteins was performed as an independent approach to confirm the placement of the strains within the genus. Phylogenetic analysis was also performed using bcgTree v1.2.1 (Ankenbrand and Keller, 2016), which reconstructs phylogenies based on 107 conserved bacterial proteins. The analysis included 54 publicly available *Legionella* genomes retrieved from NCBI, together with the two novel strains (Supplementary Data Sheet 1). The bcgTree pipeline integrates hmmsearch v3.3 (Eddy, 2011), MUSCLE v3.8.1551 (Edgar, 2004), Gblocks v0.91b (Talavera and Castresana, 2007), RAxML v8.2.12 (Stamatakis, 2006) and Prodigal v2.6.3 (Hyatt et al., 2010), and generated a best scoring maximum-likelihood tree with branch support values calculated from 100 bootstrap replicates. These approaches are based on conserved core genomic elements and are therefore equivalent to core-genome phylogenetic analyses.

Overall Genome Related Indices (OGRIs) were determined to assess genomic relatedness between the proposed novel taxa and currently described species of the genus *Legionella*. ANI values were calculated using the OrthoANIu algorithm implemented in EZBioCloud platform (Yoon et al., 2017). dDDH values were estimated using the Genome–Genome Distance Calculator (GGDC 4.0, DSMZ), applying formula d4 (also referred to as GGDC formula 2; Meier-Kolthoff et al., 2013).

Genome-based species validation was further performed using the Type (Strain) Genome Server (TYGS, DSMZ; Meier-Kolthoff and Göker, 2019; Meier-Kolthoff et al., 2022) providing comparison against type material using a standardized genome-based taxonomic framework. Draft genome assemblies were submitted to TYGS where pairwise genome comparisons were computed using the Genome BLAST Distance Phylogeny (GBDP; Meier-Kolthoff et al., 2013) approach and dDDH formulas d0, d4, and d6. The best-matching type strains, dDDH values (with 95 % CI), G+C differences and the GBDP phylogeny were retrieved from the TYGS report.

### 2.5 Phenotypic analysis

Phenotypic characterization was performed to identify diagnostic traits and to complement genome-based evidence in accordance with the polyphasic approach required for the description of novel species. This included the assessment of optimum growth temperature, the nutritional and oxygen requirements, and colony appearance.

The strains were incubated on BCYEα agar at 25, 28, 30, 35, 37, 40, 42 C for 7 days. Nutritional requirements were evaluated at their respective optimum temperatures on BCYEα, BCYEα-cys, BCYE supplemented with Cefamandole, Polymixin, and Anisomycin (BMPA, Thermo Fischer Diagnostics, Germany), GVPC, Chocolate Enriched Agar, Columbia Blood Agar, and Tryptone Soya Agar (TSA; bioMèrieux, Marcy-l'Étoile, France) for 7 days. Growth in Aces Yeast Extract (AYE) liquid medium was also assessed within 24 h.

Growth under microaerophilic conditions (O_2_ 5%−15 %) was evaluated using the GasPak EZ Campy Gas Generating Pouch System (Becton Dikinson, Franklin Lakes, NJ, USA).

All experiments were performed in three independent experiments.

Autofluorescence under long-wave ultraviolet light (wavelength of 365 nm) was also evaluated. Gram staining, acid-fast staining and motility (hanging-drop method on concave microscope slides; VWR, International, Spain) were performed as previously described (Gerhardt et al., 1994).

Morphological features and the presence of flagella were investigated by transmission electron microscopy (TEM) using an FEI Tecnai 12 electron microscope (Thermo Fisher, Schwerte, Germany) and images were recorded with an FEI Ceta CCD camera. Both strains were cultured carefully to avoid flagellar loss during sample handling. Strain CNM-1927-20^T^ was grown in AYE medium at 37 C with gentle agitation (80 rpm) for 2 days, while strain CNM-4043-24^T^, which cannot grow in liquid medium, was cultured on BCYEα agar at 30 C for 3 days, and a single colony was then carefully resuspended in PBS. Both suspensions were deposited directly onto 300-mesh copper grids (CF300-Cu, Electron Microscopy Sciences) coated with a collodion support film and negatively stained with 2% (W/V) aqueous uranyl acetate for 1 min. For each strain, three independent grids were prepared, and ten microscopic fields per grid were examined.

### 2.6 Biochemical features

Biochemical and enzymatic tests were carried out to identify differential traits useful for distinguishing the novel strains from their closest phylogenetic relatives.

Biochemical characterization of the strains studied was performed using the miniaturized API 20 NE and API ZYM systems according to the manufacturer's instructions (bioMérieux), at their respective optimum temperatures. Cytochrome C oxidase activity was determined using an oxidase reagent kit (bioMérieux). Additional enzymatic activities not included in the miniaturized test systems were assayed separately. Catalase activity was determined according to (Cowan et al. 1993) by the Identification and Characterization Services of the Spanish Type Culture Collection (CECT, Universitat de València, Paterna, Spain). Hippurate hydrolysis was tested using a 0.5 McFarland bacterial suspension prepared in 1 % PBS containing sodium hippurate (96%, Fischer Scientific, USA). The reaction mixture was incubated at 37 C for 4 h and revealed with ninhydrin reagent (Scharlab, S.L., Barcelona, Spain); the appearance of a blue color within 10 min was considered a positive result. Fermentation of glucose and lactose was assessed using bromocresol purple assay and β-lactamase production was evaluated using nitrocefin disk (Remel Inc., Thermo Fischer, USA). Positive and negative controls specified by manufacturers were included in all assays.

Antimicrobial susceptibility testing was performed to compare the phenotypic resistance profile with *in silico* genomic predictions. Susceptibility to clinically relevant antibiotics, including a macrolide (azithromycin), a fluoroquinolone (levofloxacin), rifampicin, a tetracycline (doxycycline) and pleuromutilin (lefamulin). Two methods were used: broth microdilution (BMD) protocol following Sewell et al. (2025) and the gradient diffusion method (ETEST^®^, bioMérieux).

For BMD, antibiotics were serially diluted in 96-well microtiter plates in AYE medium at the following ranges: 0.08–4 μg/mL for azithromycin and lefamulin, 0.125–64 μg/mL for doxycycline, 0.0025–0.125 μg/mL for levofloxacin and 0.00025–0.125 μg/mL for rifampicin. Bacterial suspensions were prepared to a 1.0 McFarland standard and subsequently diluted to 3–4 × 106 CFU/mL. The wells were inoculated with 100 μL of bacterial suspension and incubated at 37 ± 2 C with shaking at 160 rpm in a humidified incubator. Minimum inhibitory concentration (MIC) values were recorded after 48 h. The control strains were included in the trial (*Legionella pneumophila* NCTC 11192, NCTC 11286 and 23s rRNA mutant; Sewell et al., 2025).

ETEST^®^ were performed by swabbing 0.5 McFarland suspensions onto BCYEα agar and applying antibiotic gradient strips of azithromycin, doxycycline, levofloxacin, and rifampicin. Plates were incubated at 37 ± 2 C for 72 h in a humidified atmosphere. *Staphylococcus aureus* ATCC 29213 was included as a quality. All susceptibility tests were performed in triplicate in three independent experiments.

Cellular fatty acid (CFA) analysis was included as a chemotaxonomic criterion to support species differentiation within the genus. Fatty acid methyl ester (FAME) profiles were determined by the Identification and Characterization Services of CECT using the MIDI Microbial Identification System (Sasser, 1990). Bacterial biomass was obtained under the growth conditions described above, and fatty acid methyl ester (FAME) profiles were determined by gas chromatograph using an Agilent 6850 system. Data were analyzed using the MIDI Sherlock Microbial Identification System software with the CLIN6 method (MIDI, 2008).

### 2.7 Quantification and statistical analysis

For experiments performed in multiple independent replicates, results are expressed as means ± standard deviation (SD) or with 95 % confidence interval (CI), as appropriate.

## 3 Results

### 3.1 Environmental origin and isolation context

The two strains characterized in this study were recovered during routine environmental surveillance of independent EWS located in the Comunidad Foral de Navarra, Spain. Both systems are representative of EWS subjected to continuous thermal regulation and chemical disinfection, conditions known to influence *Legionella* persistence and population structure.

Although both strains originated from hot water distribution systems, the facilities differed in operational parameters, including disinfection strategy and hydraulic configuration. These environmental variables are known to shape microbial community composition and may impose selective pressures related to oxidative stress tolerance, membrane stability and metabolic adaptation. The recovery of two genomically distinct *Legionella* strains from such systems underscores the ecological heterogeneity present within EWS and provides a contextual framework for interpreting the genomic and phenotypic divergence described below.

### 3.2 Presumptive identification based on 16S rRNA and *mip*

To obtain an initial assessment of taxonomic placement, near full-length 16S rRNA (~1,400 bp) and partial *mip* gene (~ 600 bp) corresponding to the standard fragment described by Ratcliff et al. (1998) were amplified and compared with those of all validly described *Legionella* species.

For strain CNM-1927-20^T^, 16S rRNA gene sequence similarities ranged from 91.81 to 97.93% relative to recognized species, with *L. gresilensis* representing the closest related species. Strain CNM-4043-24^T^ showed similar values between 91.92 and 95.67%, with *L. geestiana* as the closest relative (Supplementary Table S1). Although both strains clearly affiliated with the genus, none reached the ~98.65% 16S rRNA gene sequence similarity proposed for species delineation (Kim et al., 2014).

Analysis of the *mip* gene, a marker widely used for *Legionella* species discrimination, revealed even greater divergence. Relative to other *Legionella* strains, CNM-1927-20^T^ showed sequence similarities ranged from 68.66 to 91.51%, whereas CNM-4043-24^T^ displayed values between 66.91 and 76.81% (Supplementary Table S2). The closest relatives identified based on *mip* gene similarity were consistent with those obtained using the 16S rRNA gene. These values are well below the ~95%−96 % sequence similarity threshold generally considered for species delineation based on the *mip* gene (Fry et al., 2007; Ratcliff et al., 1998), suggesting that both strains represent distinct taxonomic entities.

**Figure 1:**
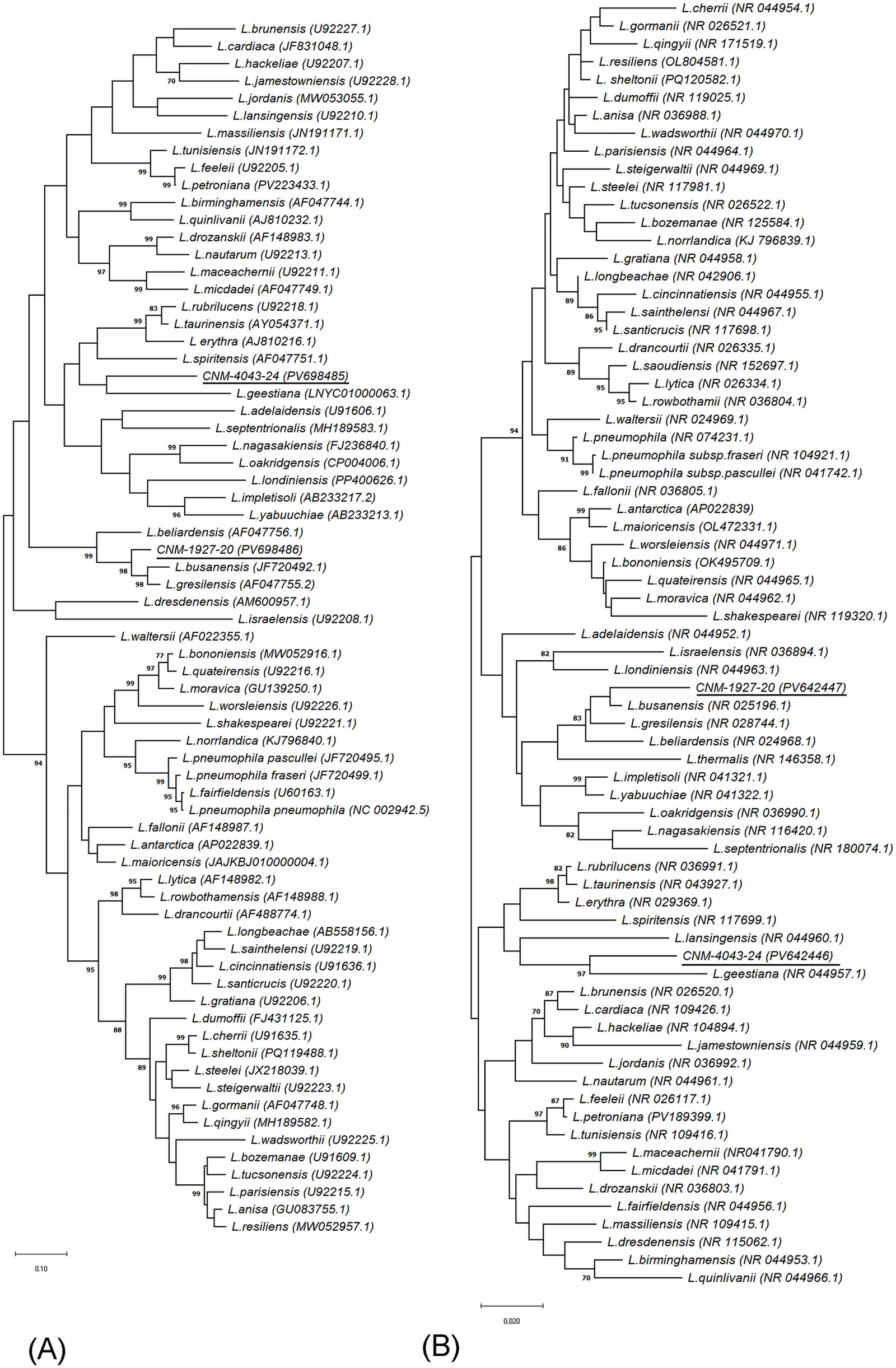
Phylogenetic analysis based on 16S rRNA gene sequences **(A)** and *mip* gene sequences **(B)**. Trees were inferred using the maximum likelihood method, with bootstrap support values calculated from 1,000 replicates shown next to the branches, bootstrap values below 70 % were omitted. GenBank accession numbers are indicated in parentheses. Novel proposed species in this study are underlined. The scale bar represents the number of nucleotide substitutions per site.

Maximum-likelihood phylogenetic trees inferred from both 16S rRNA and *mip* sequences consistently placed the two strains within the *Legionella* clade while resolving them as independent branched. Strain CNM-4043-24^T^ clustered within the *Legionella geestiana* species clade, whereas strain CNM-1927-20^T^ was positioned in proximity to the *Legionella busanensis* and *Legionella gresilensis* species clades, although clearly separated from all currently described species (Figure 1). Bootstrap values below 70% were not displayed. The topologies obtained from both loci were congruent and did not indicate close affiliation with any single recognized taxon.

To improve the robustness of phylogenetic inference, additional analyses were performed using IQ-TREE with 1,000 ultrafast bootstrap (UFBoot) and SH-aLRT replicates. Low-support nodes (<70 %) were not displayed. The resulting topology was consistent with that obtained, with both strains clearly resolved within the genus *Legionella* as independent lineages. These results confirm that the phylogenetic placement of the isolates is stable and not affected by the inference method (Supplementary Figure S1).

Taken together, single-gene phylogenies provided preliminary evidence of taxonomic novelty and supported subsequent genome-scale analyses to resolve their evolutionary position with higher resolution.

### 3.3 Genome features and assembly quality

Whole-genome sequencing was performed to characterize the genomic architecture of strains CNM-1927-20^T^ and CNM-4043-24^T^ and to assess their relationship to described members of the genus *Legionella*. High-quality draft assemblies were obtained for both strains, with estimated completeness values above 97% and minimal contamination (<1%), supporting the reliability of downstream comparative analyses.

Strain CNM-1927-20^T^ possesses a genome of 3,346,181 bp distributed across 33 contigs (>1,000 bp), with sizes ranging from 1,000 to 532,143 bp (mean contig length = 101,400 bp), an N50 of 203,734 bp, L50 of 5 and a G+C content of 35.52 mol%. The genome encodes 2,979 predicted coding sequences, together with 4 rRNA genes, 42 tRNA genes and 1 tmRNA gene. In contrast, strain CNM-4043-24^T^ has a slightly smaller genome of 3,019,141 bp assembled in 55 contigs (>1,000 bp), ranging from 1,000 to 404,651 bp (mean contig length = 54,900 bp), with an N50 of 114,967 bp, L50 of 8 and a G+C content of 48.72 mol%. This genome contains 2,596 predicted coding sequences, 3 rRNA genes, 42 tRNA genes and 1 tmRNA gene.

While genome sizes fall within the range commonly reported for *Legionella* species, the G+C content of CNM-4043-24^T^ lies toward the upper boundary of values described for the genus, highlighting the genomic heterogeneity encompassed within environmental representatives. Despite this divergence, both genomes retain key genetic features characteristic of *Legionella*, including genes associated with intracellular survival and conserved core metabolic pathways.

Comparative genome screening did not detect plasmids or acquired antimicrobial resistance determinants in either strain. Functional annotation revealed broadly conserved core functional categories across both genomes (see Section 3.7 and Supplementary Figure S3), although variation in accessory gene content was observed between the two strains, consistent with their phylogenetic separation. Together, these genomic features indicate that the strains share fundamental genomic characteristics with established members of the genus while displaying substantial divergence at the nucleotide level.

This genomic framework provided the basis for subsequent phylogenomic reconstruction and genome-wide relatedness analyses aimed at resolving their taxonomic placement.

### 3.4 Genome-based relatedness indices

To quantitatively assess genomic relatedness, ANI and dDDH values were calculated. Genome-based relatedness indices were calculated against representative *Legionella* species selected on the basis of their position in the genome-based phylogenetic tree, prioritizing taxa clustering within the same clade or placed in close phylogenetic proximity to the proposed novel taxa (Supplementary Tables S3A, S3B). Additionally, ANI comparisons were also extended to all validly described *Legionella* species showing ≥95% 16S rRNA gene sequence similarity with either CNM-4043-24^T^ or CNM-1927-20^T^ (Supplementary Table S3E).

For strain CNM-1927-20^T^, the highest ANI values were observed with *L. gresilensis* (85.01%), followed by *L. busanensis* (84.41%) and *L. beliardensis* (76.91%). For strain CNM-4043-24^T^, the highest ANI value was obtained with *L. dresdenensis* (70.37%), followed by *L. birminghamensis* (69.58%) and *L. quinlivanii* (69.24%). All values were substantially below the widely accepted species delineation threshold of 95%−96%, indicating pronounced nucleotide-level divergence relative to their closest described neighbors.

ANI values between CNM-1927-20^T^ and CNM-4043-24^T^ were similarly low (68.83%), further supporting their recognition as two independent genomic entities rather than variants of a single novel taxon.

Additional ANI comparisons with all species sharing ≥95% 16S rRNA gene sequence similarity likewise yielded values below the accepted species delineation threshold, further supporting the gemomic distinctiveness of both strains (Supplementary Table S3).

Digital DNA–DNA hybridization estimates corroborated this pattern, with all calculated dDDH values ranging between approximately 12 and 40% depending on the comparison and consistently remaining well below the 70% threshold used for species delineation. For CNM-4043-24^T^, dDDH values relative to its closest phylogenetic neighbors did not exceed 13.2%, underscoring its marked genomic divergence (Supplementary Tables S3C, S3D).

Genome-based validation using TYGS server confirmed that both isolates fall well below the boundary defined by dDDH. For CNM-4043-24^T^, the highest dDDH (formula d4) against any type strain was 28.7% (95% CI 26.3–31.2; *Pseudomonas graminis*), and the highest d4 to a *Legionella* type strain was 23.5% (95% CI 21.2–25.9; *Legionella lytica*); with a G+C difference of 9.13 mol%.

For CNM-1927-20, the highest dDDH (d4) was 30.1% (95% CI 27.7–32.6) to *Legionella gresilensis*, with a G+C difference of 0.54 mol%. As all dDDH (d4) values are far below the 70% species threshold, the TYGS results support the status of both isolates as potential novel species within the genus.

Taking together, these genome-wide relatedness metrics quantitatively confirm that both strains represent deeply divergent yet phylogenetically coherent members of the genus *Legionella*.

### 3.5 Phylogenomic placement

**Figure 2:**
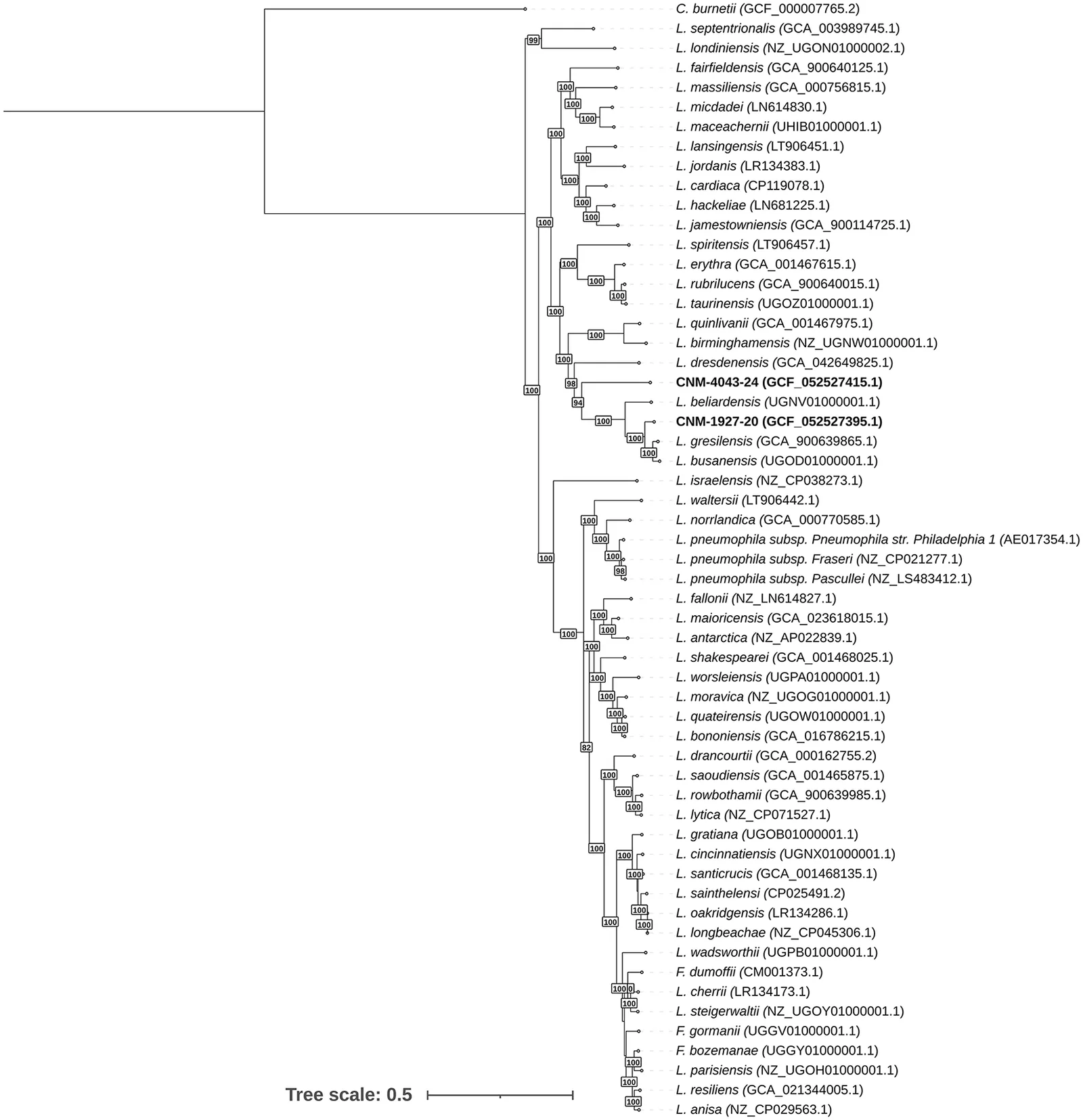
Maximum-likelihood phylogenomic tree shows the relationships between strain CNM-1927-20^T^, strain CNM-4043-24^T^, and 54 other representative *Legionella* genomes, as well as *Coxiella burnetii* as an outgroup. The tree was inferred using bcgTree software, as described in Material and Method section. Bootstrap consensus trees were calculated from 100 replicates and are shown next to the branches. Bootstrap values below 70% were omitted. Branch lengths represent the number of substitutions per site and indicate the inferred evolutionary divergence among taxa. GenBank accession ID is given in parentheses. Novels described species in this study are highlighted in bold.

To resolve the evolutionary position of the two strains, genome-scale phylogenetic analyses were performed using conserved core genes. Phylogenomic reconstruction based on 107 conserved bacterial proteins (bcgTree) consistently placed strains CNM-1927-20^T^ and CNM-4043-24^T^ within the *Legionella* clade with strong bootstrap support (Figure 2). Both strains formed independent and well-supported branches clearly separated from all currently described species included in the analysis. Strain CNM-1927-20^T^ clustered with *L. gresilensis*, and *L. busanensis*, forming a well-supported clade within the genus. In contrast, strain CNM-4043-24^T^ also grouped within this broader lineage and clustered most closely with *L. gresilensis* and *L. busanensis*, being part of the same clade that includes CNM-1927-20^T^. Although phylogenetically affiliated with these data, strain CNM-4043-24^T^ remained clearly differentiated from these taxa based on genome-based relatedness indices.

To further support these findings, a core genome multilocus sequence typing (cgMLST) approach based on 436 conserved loci was implemented. The resulting maximum-likelihood tree reproduced the phylogenetic structure observed in the protein-based analysis, with both strains consistently resolved as discrete genomic entities within the genus. The concordance between independent phylogenomic frameworks reinforces the robustness of their evolutionary placement (Supplementary Figure S2).

The phylogenetic reconstructions based on core genomic loci (cgMLST) and conserved proteins (bcgTree) yielded congruent topologies, supporting the robustness of the inferred evolutionary relationships.

Despite the elevated G+C content observed in CNM-4043-24^T^, genome-scale phylogenetic reconstruction unequivocally supports its inclusion within *Legionella*. The consistent clustering within the genus across independent datasets indicates deep intrageneric divergence rather than genus-level separation.

These results collectively demonstrate that both strains represent previously unrecognized evolutionary units embedded within the genomic landscape of *Legionella*.

### 3.6 Functional annotation and COG classification

To facilitate comparison between strains, the distribution of the most abundant COG functional categories was analyzed. Both genomes showed a similar overall functional profile, with “Unknown function” representing the largest category (Supplementary Figure S3).

Core cellular functions such as translation, cell wall/membrane biogenesis, and replication/repair were consistently represented in both genomes, reflecting the conservation of essential biological processes within the genus *Legionella*.

Minor differences were observed in metabolic categories, particularly those related to amino acid transport and carbohydrate metabolism, suggesting some degree of functional divergence between the strains.

Overall, this analysis highlights the conservation of core functional categories alongside variability in accessory functions, supporting the genomic differentiation observed between the strains.

### 3.7 Functional genomic features associated with environmental persistence

Comparative genomic analysis was performed to explore functional traits potentially associated with environmental persistence and intracellular survival. Both genomes encoded core genetic determinants typically conserved within the genus *Legionella*, including components of secretion systems implicated in host interaction and intracellular replication. The presence of these conserved systems reinforces their placement within the genus and suggests maintenance of canonical ecological strategies centered on protozoan hosts.

Initial screening using ARIBA and AMRFinderPlus under default thresholds did not identify acquired antimicrobial resistance determinants or an expanded virulence gene repertoire. To account for potential sequence divergence, additional analyses were performed with ABRicate v1.4.0 using relaxed thresholds (≥60% identity and ≥80% coverage).

Under these thresholds, several virulence-associated homologs were detected in both genomes, primarily corresponding to conserved components of intracellular survival and secretion system. Most of these homologs were shared between the two strains, although some differences in accessory gene content were observed. Comparative analysis including closely related *Legionella* species revealed that the number of virulence-associated homologs detected in strains CNM-1927-20^T^ (53 genes) and CNM-4043-24^T^ (38 genes) falls within the range observed across the genus (49–67 genes). Notably, strain CNM-4043-24^T^ displayed a reduced number of detected homologs, consistent with its higher genomic divergence.

Overall, these results indicate that both strains retain the core virulence-associated repertoire typical of *Legionella* species without evidence of an expanded or atypical virulome.

Screening for antimicrobial resistance revealed only a limited number of low-similarity homologs (1–3 per genome), which were also present in related species. These likely represent conserved domain rather than acquired antimicrobial resistance determinants. Accordingly, no evidence of acquired antimicrobial resistance was observed in either genome. No plasmids were detected.

Comparative virulome and resistome profiles across all analyzed genomes were broadly consistent and represented as a heatmap (Supplementary Figure S4).

Despite overall conservation of core functions, lineage-specific genomic features were observed. In CNM-1927-20^T^, a predicted S-adenosylmethionine-dependent methyltransferase annotated as a potential type IV secretion system effector was identified. Although functional validation was beyond the scope of this study, such effectors may contribute to modulation of host–pathogen interactions within protozoan reservoirs.

The combination of conserved intracellular survival machinery and divergent accessory genomic content illustrates a pattern of genomic plasticity within environmental *Legionella*. Core ecological strategies appear to be retained, while peripheral genomic regions diversify, potentially facilitating adaptation to localized physicochemical conditions in engineered water systems. These results align with the physiological and chemotaxonomic differences described above and provide a functional dimension to the genomic divergence observed between the two strains.

### 3.8 Phenotypic and physiological characterization

Beyond genome-level divergence, both strains exhibited distinct physiological profiles consistent with ecological differentiation within EWS.

Strains CNM-1927-20^T^ and CNM-4043-24^T^ were Gram-negative, motile rods requiring L-cysteine for growth, consistent with established characteristics of the genus *Legionella*. Growth occurred between 30 C and 37 C for both strains; however, their optimal temperatures differed, with CNM-1927-20^T^ showing optimal growth at 35 C and CNM-4043-24^T^ at 30 C. Growth was also observed under microaerophilic conditions in both strains. These differences, although moderate, may reflect distinct thermal adaptation profiles within EWS.

A notable physiological distinction was the ability of CNM-1927-20^T^ to grow in liquid AYE medium, whereas CNM-4043-24^T^ failed to proliferate under the same conditions. This differential growth capacity may be due to variations in metabolic flexibility or stress tolerance under planktonic conditions. Additionally, growth occurred only on BCYEα, BMPA, and GVPC media, which indicates the L-cysteine requirements. Additionally, catalase activity was detected in CNM-1927-20^T^ but not in CNM-4043-24^T^, indicating potential differences in oxidative stress response mechanisms. Given that both strains were recovered from systems subjected to chemical disinfection, variation in oxidative stress tolerance may represent adaptive divergence associated with local environmental pressures.

Both strains were negative for oxidase, urease, nitrate reduction, and carbohydrate assimilation, catalase activity was detected only in CNM-1927-20^T^ (Table 1), consistent with the limited metabolic repertoire characteristic of environmental *Legionella* species. However, subtle differences in enzymatic activity profiles were observed in API ZYM assays (Supplementary Table S4), further supporting their phenotypic separation.

**Table 1:** Key characteristics of CNM-1927-20^T^ and CNM-4043-24^T^ and related species.

Activities	CNM-1927-20^T^	CNM-4043-24^T^	*L. beliardensis* (Montbéliard A1T)	*L. gresilensis* (Gréoux 11 D13T)	*L. busanensis* (K9951T)	*L. birminghamensis* (1407-AL-H)	*L. quinlivanii* (CCUG 31234 A)
Catalase	+	–	+	+	+	+	+
Urease	–	–	–	–	NR	–	–
Hipurate hydrolysis	–	–	–	+	+	–	–
Oxidase	–	–	+	+	+	V	–
β-lactamase production	–	–	+	+	–	+	–
Gelatinase	–	–	+	+	+	+	+
Glucose fermentation	–	–	–	–	NR	–	–
Autofluorescence	W	W	NR	NR	–	+	+

The characteristics of the related species were taken from reported literature: L. beliardensis (Lo Presti et al., 2001), L. gresilensis (Lo Presti et al., 2001), L. busanensis (Park et al., 2003); L. birminghamensis (Wilkinson et al., 1987); L. quinlivanii (Benson et al., 1989). The accession numbers are indicated in parentheses. +, positive; –negative; W, weakly positive; NR, not reported; V, variable.

Antimicrobial susceptibility testing revealed patterns consistent with environmental *Legionella* species. Both strains were susceptible to macrolides and fluoroquinolones, including erythromycin and ciprofloxacin, which remain the primary therapeutic options for legionellosis. No resistance to rifampicin was detected (Supplementary Table S5). These phenotypic results are concordant with genomic screening, which did not identify acquired antimicrobial resistance determinants in either genome.

**Figure 3:**
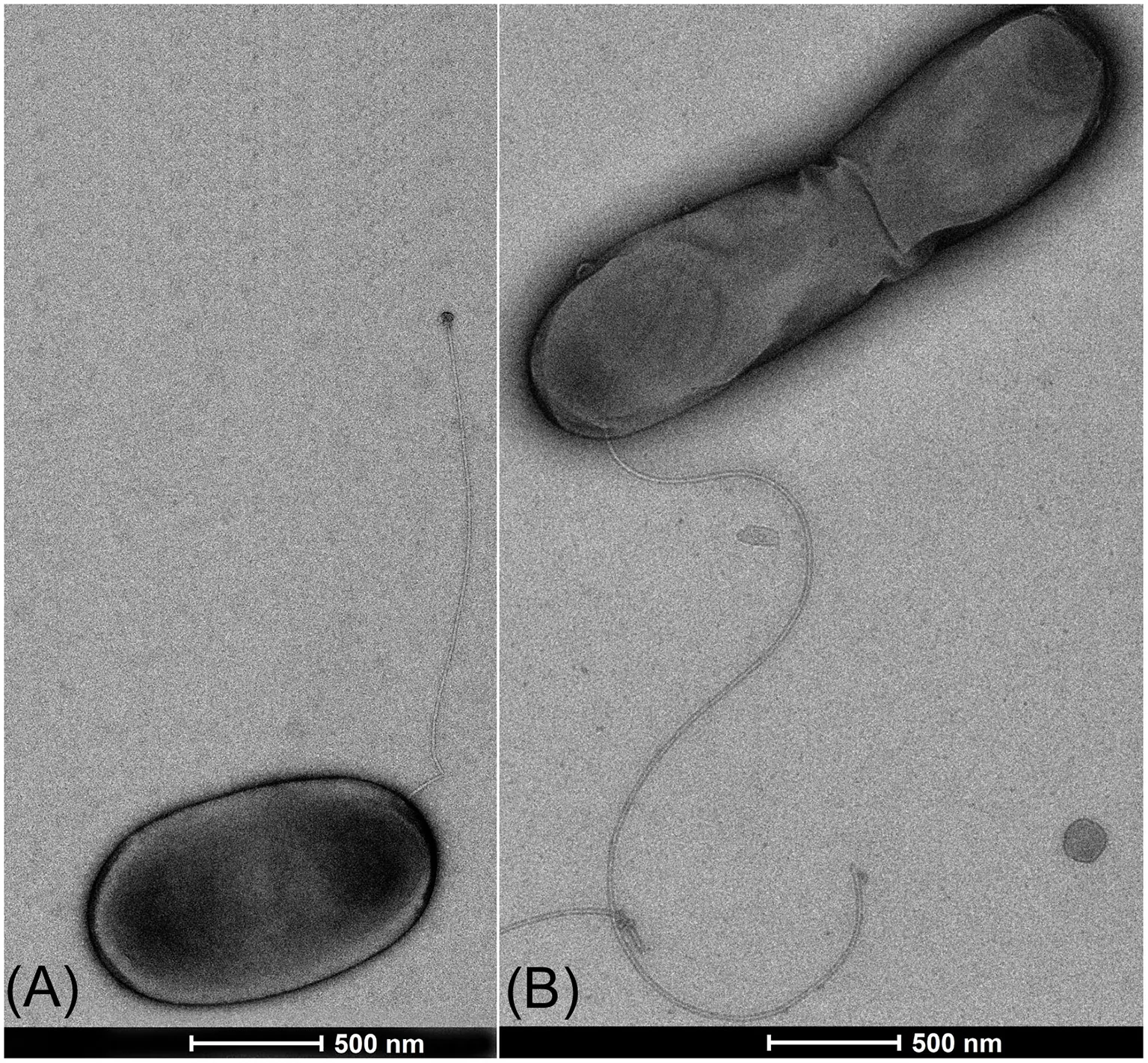
Negative staining by transmission electron microscopy (TEM) images of CNM-1927-20^T^ **(A)** and CNM-4043-24^T^ **(B)**.

**Figure 4:**
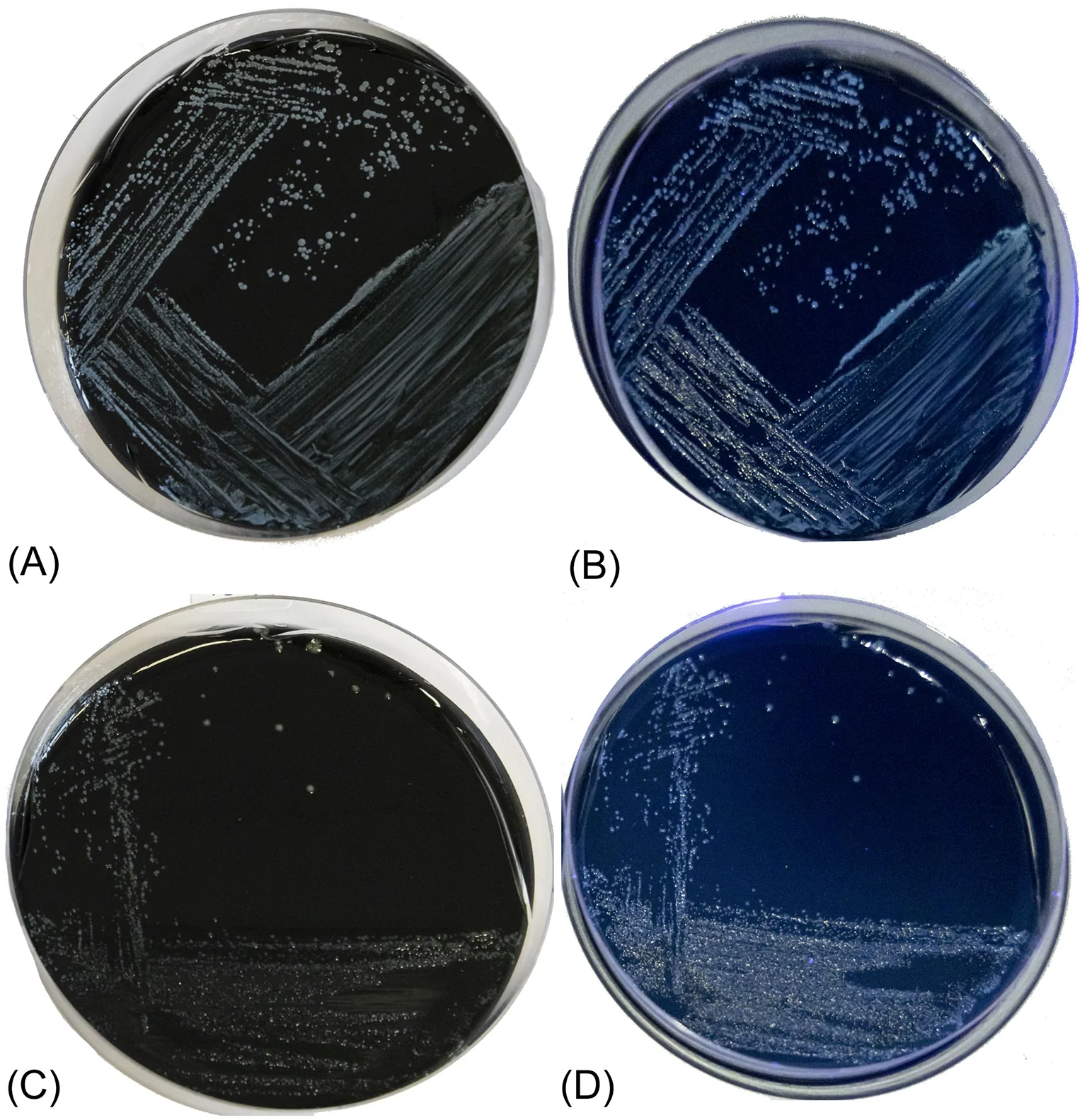
Strains CNM-1927-20^T^ **(A)** and CNM-4043-24^T^ **(C)** cultured on a BCYEα plate for 72 h of strain. Autofluorescence under UV light (365 nm) of strain CNM-1927-20^T^ **(B)** and CNM-4043-24^T^ **(D)**.

Transmission electron microscopy revealed a single polar flagellum in both strains, confirming motility and reinforcing their classification within the genus (Figure 3). Neither strain exhibited autofluorescence under long-wave UV light, distinguishing them from certain related species (Figure 4).

Taken together, these physiological and morphological features demonstrate that the two strains, while sharing core traits typical of *Legionella*, display reproducible phenotypic differences that align with their genomic separation. The observed variation in growth characteristics and oxidative stress response capacity may reflect ecological specialization within distinct EWS contexts.

### 3.9 Chemotaxonomic features

Analysis of CFA composition revealed marked differences between the two strains, consistent with their genomic divergence and suggestive of distinct physiological strategies (Table 2).

**Table 2:** Cellular fatty acids (CFA) composition of CNM-1927-20^T^ and CNM-4043-24^T^ and selected closely related species belonging in the same clade.

CFA characteristics	CNM-1927-20^T^	CNM-4043-24^T^	*L. gresilensis* (*Gréoux 11 D13^*T*^)*	*L. busanensis* (K9951^T^)^*^	*L. beliardensis* (Montbéliard A1^T^)	*L. birminghamensis* (1407-AL-H)^#^	*L. dresdenensis* (W03-356^T^)	*L. quinlivanii* (CCUG 31234 A)
Saturated								
C14:0	0.5	0.5	NR	NR	NR	NR	NR	0.8
C15:0			NR	1.8	NR	3	NR	2.7
C16:0	11.1	6	9	NR	20	13	NR	9.3
C17:0	2.1	2.7	NR	2.9	NR	TR	NR	2.4
C18:0	4.3	1.4	NR	3.5	11	TR	NR	NR
C19:0	0.8	0.8	NR	NR	NR	NR	NR	NR
C20:0	2.3	0.5	NR	0.6	NR	TR	NR	0.4
Unsaturated								
C14:1 ω-5c	0.6	0.6	NR	1.3	NR	NR	NR	0.6
C15:1 ω-6c	2.8	6	NR	5.1	NR	TR	NR	5.9
C16:1 ω-7c			33	45.1	31	TR	24.4	26.5
C16:1 ω-7c/C16:1 ω-6c (Σ)	48.6	23	NR	NR	NR	11	NR	NR
C18:1			NR	NR	15		NR	NR
Methyl branched (Iso. anteiso)								
C15:0 anteiso-	5.52	5.38	NR	1.9	NR	30	15.7	21.2
C16:0 anteiso-			NR	9.4	NR	NR	NR	NR
C17:0 anteiso-	3.83	2.70	9	1.3	9	16	8.5	10.1
C19:0 anteiso-	0.79		NR	NR	NR	NR	NR	NR
C14:0 iso-	0.71	7.06	NR	2.8	NR	TR	NR	1.9
C15:0 iso-	0.32		NR	NR	NR	NR	NR	0.8
C16:0 iso-	13.47	41.96	13	18.3	NR	14	25.3	16
C17:0 iso-	0.48		9	NR	NR	TR	NR	1
C18:0 iso-	1.74	0.65	NR	2.36	NR	NR	NR	NR
Branched-chain hydroxy								
C13:0 iso-2-OH	NR	NR	NR	NR	NR	NR	NR	0.6
C14:0 iso-3-OH	0.98	NR	NR	NR	NR	31	NR	NR
C20:0 3-OH	NR	NR	NR	NR	NR	NR	NR	NR

The accession number is indicated in parentheses. TR: minor components (relative abundance, <10). NR: not reported. (Σ): sum in feature 3.

* Means of the reported data from Park et al. (2003).

# values as relative abundance, with the most abundant component in each class considered as 100.

Strain CNM-1927-20^T^ was dominated by unsaturated fatty acids, particularly C16:1 ω-7c/ω-6c, which accounted for nearly half of the total fatty acid content. In contrast, strain CNM-4043-24^T^ displayed a predominance of iso-branched fatty acids, with C16:0 iso representing the major component. Although both profiles fall within the chemotaxonomic framework described for the genus *Legionella*, the relative abundance and distribution of specific lipid classes differed substantially between the two strains.

Membrane lipid composition plays a central role in bacterial adaptation to environmental stress, influencing membrane fluidity, permeability and resistance to thermal and oxidative challenges. Unsaturated fatty acids generally enhance membrane fluidity, whereas branched-chain fatty acids can contribute to structural stability under stress conditions. The contrasting CFA patterns observed here may therefore reflect alternative adaptive strategies associated with survival in EWS exposed to elevated temperatures and chemical disinfectants.

When compared to phylogenetically related species, both strains exhibited distinctive quantitative CFA signatures, supporting their phenotypic differentiation. Notably, the predominance of iso-branched fatty acids in CNM-4043-24^T^ distinguishes it from closely related taxa and aligns with its distinct genomic background, including its elevated G+C content.

These chemotaxonomic differences, considered alongside genomic and physiological divergence, reinforce the interpretation that the two strains represent independent evolutionary trajectories within environmental *Legionella* populations. Moreover, the observed variation in membrane composition provides a plausible mechanistic link between genomic divergence and ecological specialization in anthropogenic aquatic environments.

### 3.10 Taxonomic conclusion and species recognition

The integration of genome-scale phylogenetic reconstruction, genome-wide relatedness metrics, phenotypic characterization and chemotaxonomic profiling provides a coherent and multilayered framework for taxonomic assessment. Across independent analytical approaches, strains CNM-1927-20^T^ and CNM-4043-24^T^ consistently resolved as distinct genomic entities embedded within the genus *Legionella*.

Phylogenomic analyses robustly supported their placement within the genus while clearly separating them from all currently recognized species. Genome-based relatedness indices further confirmed this distinction, with ANI and dDDH values well below accepted species delineation thresholds relative to their closest phylogenetic neighbors. In addition, the two strains exhibited reproducible physiological and chemotaxonomic differences consistent with their genomic divergence and suggestive of ecological differentiation.

Importantly, ANI values between CNM-1927-20^T^ and CNM-4043-24^T^ were also below species-level threshold, demonstrating that they represent two independent evolutionary units rather than variants of a single novel taxon. Despite the relatively elevated G+C content observed in CNM-4043-24^T^, genome-scale phylogenetic reconstruction and conserved functional features unequivocally support its inclusion within the genus *Legionella*.

Taken together, the combined genomic, phenotypic, and phylogenetic evidence fulfills contemporary standards for species delineation in prokaryotic taxonomy. We therefore conclude that strains CNM-1927-20^T^ and CNM-4043-24^T^ represent two novel species within the genus *Legionella*, for which the names *Legionella pelazae* sp. nov. and *Legionella navarrensis* sp. nov. are proposed.

### 3.11 Species descriptions

#### 3.11.1 Description of *Legionella pelazae* sp. nov.

*Legionella pelazae* (pe.la'zae. N.L. gen. n. *pelazae*, in honor of the Spanish microbiologist Carmen Pelaz [† 2025], who pioneered the study of legionellosis in Spain).

Cells are Gram-negative and Ziehl-Neelsen negative, microaerobic, rod-shaped, and flagellated. Growth occurs on BCYEα, BMPA, and GVPC agar at temperatures between 30 and 37 C, with optimal growth at 35 C. It is negative for gelatinase, oxidase, urease and hippurate hydrolysis, and positive for catalase activity. It is non-fermentative, and nitrate reduction is negative. The genome size is 3.35 Mb, with a DNA G+C content of 35.52 mol%. The cellular fatty acids profile is dominated by unsaturated fatty acids. The type strain, CNM-1927-20^T^ (BioSample accession SAMN48408482) was isolated on 2 March 2020 from a spa facility in the Comunidad Foral de Navarra, Spain. The GenBank accession number for the *mip* gene sequence is PV698486, and for the 16S rRNA gene sequence is PV642447. The GenBank accession number for the whole genome shotgun Project is PRJNA1260987. This strain is deposited as strain type in the Spanish Type Culture Collection (CECT, accession number CECT 31282) and Culture Collection University of Gothemburg (CCUG, accession number CCUG 78397^T^).

#### 3.11.2 Description of *Legionella navarrensis* sp. nov.

*Legionella n*avarrensis (na.var.ren'sis. N.L. fem. adj. *navarrensis*, pertaining to Navarra, Spain, where the organism was isolated).

Cells are Gram-negative and Ziehl-Neelsen negative, microaerobic, rod-shaped and flagellated. Growth occurs on BCYEα, BMPA and GVPC agar at temperatures between 30 and 37 C with optimal growth at 30 C. It is negative for catalase, oxidase, urease and hippurate hydrolysis, and positive for gelatinase activity. It is non-fermentative, and nitrate reduction is negative. The genome size is 3.02 Mb, with a DNA G+C content of 48.72 mol%. The cellular fatty acid profile is dominated by branched-chain fatty acids. The type strain CNM-4043-24^T^ (BioSample accession SAMN48404767) was isolated on 14 February 2024 from a rural tourism accommodation in a town in the Comunidad Foral de Navarra, Spain. The GenBank accession number for the *mip* and 16S rRNA gene sequences are PV698485 and PV642446, respectively. The GenBank accession number for the whole genome shotgun project is PRJNA1260922. This strain is deposited as strain type in the Spanish Type Culture Collection (CECT, accession number CECT 31283) and Culture Collection University of Gothemburg (CCUG, accession number CCUG 78398^T^).

## 4 Discussion

The present study expands the evolutionary and ecological framework of the genus *Legionella* through the characterization of two environmental strains recovered from independent EWS. By integrating single-gene phylogenies, genome-scale reconstruction, genome-based relatedness metrics, phenotypic analyses and chemotaxonomic profiling, we demonstrate that strains CNM-1927-20^T^ and CNM-4043-24^T^ represent two previously unrecognized species embedded within the genomic landscape of the genus.

Traditional identification of *Legionella* species based on partial gene sequencing, such as 16S rRNA and *mip*, is commonly used for preliminary taxonomic assignments. Species delineation based on these markers is generally guided by similarity thresholds of ≥98.7–99% for 16S rRNA gene sequences and ≥95%−96% for *mip* (Fry et al., 2007; Ratcliff et al., 1998); however, these loci often lack sufficient resolution to accurately capture deep genomic divergence and may underestimate species-level diversity within the genus (Kim et al., 2014). Consequently, genome-based approaches provide a more robust framework for species delineation. Genome-wide comparisons revealed contrasting degrees of divergence between both strains. CNM-1927-20^T^ exhibited its highest ANI value with *L. gresilensis* (85.01%), consistent with clear species-level separation while remaining within the diversity spectrum typically observed among established *Legionella* clades. In contrast, CNM-4043-24^T^ displayed a maximum ANI of 70.37% relative to *L. dresdenensis*, with similarly low values against other neighboring taxa (Rodriguez et al., 2021). Expanding ANI comparisons across a broader representation of the genus did not alter the taxonomic interpretation, as all values remained well below the species boundary.

Such levels of nucleotide divergence are uncommon within most bacterial genera and position CNM-4043-24^T^ among the most genomically distinct environmental representatives described to date. Nevertheless, phylogenomic reconstruction based on conserved core proteins robustly supports its placement within *Legionella* genus, illustrating the considerable evolutionary breadth encompassed by the genus.

However, recent large-scale analyses of prokaryotic genome diversity have demonstrated that while an ANI threshold of ~95%−96% robustly delineates species boundaries, intrageneric ANI ranges are highly variable and lineage-dependent (Murray et al., 2021). In genera characterized by ancient diversification and ecological radiation, members may display reduced pairwise ANI values while still forming coherent and well-supported phylogenomic clusters (Konstantinidis and Tiedje, 2005).

In the present case, the low ANI values are accompanied by limited alignment coverage (approximately 20%), consistent with extensive genomic divergence rather than taxonomic misplacement. The restricted proportion of shared genomic regions further suggests long-term evolutionary separation accompanied by extensive accessory genome remodeling. Importantly, phylogenomic reconstruction based on conserved core loci robustly situates CNM-4043-24^T^ within the *Legionella* clade, supporting its inclusion within the genus despite its pronounced nucleotide-level divergence.

Although ANI does not capture genome rearrangements, its use in combination with phylogenomic analyses provides a robust framework for species delineation.

The genus *Legionella* is characterized by marked genomic plasticity, with genome sizes ranging from approximately 2.37–4.88 Mb and G+C contents spanning 34.82%−50.93%, reflecting substantial evolutionary heterogeneity often associated with horizontal gene transfer (Gomez-Valero et al., 2011, 2019). Although a 95% ANI threshold is widely applied for species delineation, recent genomic studies have emphasized that genetic boundaries may vary according to lineage-specific evolutionary histories and ecological specialization (Rodriguez et al., 2021). In highly diverse genera such as *Legionella*, deep divergence may therefore reflect adaptation to discrete ecological niches rather than taxonomic displacement. Species delimitation in this group should consequently rely on an integrative framework combining genome-wide metrics, phylogenetic coherence and ecophysiological differentiation, recognizing that genetically distant populations may follow parallel evolutionary trajectories under distinct selective pressures.

This combination of genomic divergence and phylogenetic coherence is supported by comparative functional annotation analyses performed in this study. The distribution of COG functional categories revealed a largely conserved functional profile across strains, consistent with the maintenance of core functions associated with intracellular survival. Core determinants involved in host interaction and intracellular replication appear to be conserved across the genus, reflecting a shared ecological strategy linked to protozoan reservoirs (Gomez-Valero et al., 2011). In contrast, variability observed in specific functional categories may reflect diversification in accessory genomic regions, that potentially may contribute to adaptation to localized physicochemical conditions. Such a pattern is consistent with a model of evolutionary diversification driven by ecological opportunity within the constraints of an intracellular lifestyle (Cazalet et al., 2004).

The conservation of virulence-associated homologues across all analyzed genomes supports the notion that the intracellular lifestyle of Legionella relies on a stable core set of functions, while variation in accessory gene content reflects lineage-specific diversification.

The elevated G+C content observed in CNM-4043-24^T^ (48.72 mol%) expands the compositional spectrum represented in this study but remains within the broader range documented for the genus. Genome-wide analyses of *Legionella* diversity have reported G+C contents reaching approximately 51% (Gomez-Valero et al., 2019), indicating that the nucleotide composition of the genus is more heterogeneous than previously appreciated. Moreover, digital DNA–DNA hybridization comparisons revealed G+C differences of 5%−14% relative to phylogenetically neighboring taxa, consistent with substantial evolutionary separation but not incompatible with genus-level affiliation. Genome quality metrics and the absence of anomalous contigs exclude assembly artifacts as a plausible explanation. Rather than representing taxonomic displacement, the elevated G+C content is fully consistent with its stable phylogenomic placement within the genus and likely reflects lineage-specific evolutionary dynamics associated with long-term ecological specialization within engineered aquatic environments.

Single-gene phylogenies based on 16S rRNA and *mip* provided preliminary evidence of taxonomic novelty and remain highly relevant within the genus. Importantly, the congruence between phylogenetic reconstructions obtained using different inference methods further supports the robustness of the observed topology. The *mip* gene, historically central to *Legionella* species delineation, exhibited levels of divergence consistent with the genome-wide analyses, reinforcing the robustness of the taxonomic conclusions (Ratcliff et al., 1998). Importantly, the congruence between single-locus trees and phylogenomic reconstruction indicates that the observed divergence is not an artifact of genome-scale methodology but reflects genuine evolutionary separation.

Phenotypic and chemotaxonomic differentiation further support ecological divergence. Differences in optimal growth temperature, catalase activity and ability to proliferate in liquid medium suggest variation in stress tolerance and metabolic flexibility. Membrane fatty acid composition showed contrasting patterns, with CNM-1927-20^T^ dominated by unsaturated fatty acids and CNM-4043-24^T^ enriched in iso-branched lipids. Although elevated temperatures are commonly associated with increased proportions of saturated fatty acids, membrane remodeling in environmental *Legionella* is likely influenced by multiple selective pressures, including oxidative stress, disinfectant exposure and fluctuating thermal regimes. Iso-branched fatty acids and unsaturated lipids both contribute to modulation of membrane fluidity and permeability, suggesting that distinct compositional strategies may support persistence under complex physicochemical conditions in EWS.

The comparison of COG functional profiles between both strains revealed differences that may be linked to their phenotypic and ecological traits. *L. pelazae* sp. nov. exhibited a higher representation of genes associated with cell wall/membrane/envelope biogenesis and translation-related functions. This may be related to its growth optimum at higher temperatures (35 C) and the predominance of unsaturated fatty acids, which are typically associated with membrane fluidity and adaptation to thermal conditions. The presence of catalase activity in this strain may also reflect enhanced oxidative stress response capabilities, consistent with a more dynamic environmental niche such as spa facilities.

In contrast, *L. navarrensis* sp. nov. showed a relatively higher proportion of proteins assigned to unknown functions, suggesting a larger fraction of uncharacterized genetic potential. This strain also displayed distinct phenotypic traits, including gelatinase activity and a fatty acid profile dominated by branched-chain fatty acids, which are often associated with adaptation to lower temperatures and environmental persistence. Its optimal growth at 30 C may reflect adaptation to less thermally stable environments such as rural water systems.

Differences in metabolic categories, including amino acid and carbohydrate metabolism, may further support distinct ecological strategies between the strains. Additionally, variation in signal transduction and intracellular trafficking categories could indicate differences in environmental sensing and host interaction capabilities, which are key features of *Legionella* species.

Overall, the integration of functional annotation and phenotypic data suggests that both strains share core cellular functions typical of the genus, while exhibiting distinct genomic signatures that may underlie their ecological specialization and adaptation to different aquatic environments.

A more detailed characterization of mobile genetic elements (MGEs) and accessory genome dynamics would require dedicated pangenomic and structural analyses, which were beyond the scope of this taxonomic study. In this study, genome annotation was performed using Prokka for general gene prediction, complemented by functional annotation with eggNOG-mapper to explore functional variability across strains. While this approach provides an overview of functional diversification, it does not specifically target the identification of MGEs or the detailed characterization of the accessory genome. Dedicated analyses focusing on genomic islands, integrons or plasmids would be required to address these aspects in greater depth.

The public health implications of these findings warrant consideration. Environmental surveillance programs primarily target *L. pneumophila*, potentially underestimating the diversity of non-*pneumophila* species present in water distribution networks. Although neither strain harbored acquired antimicrobial resistance determinants and no expanded repertoire of virulence genes was detected, the presence of conserved intracellular survival machinery indicates maintenance of the ecological traits that define the genus. Expanding genomic reference frameworks is therefore critical for improving diagnostic resolution, refining environmental monitoring strategies and supporting epidemiological investigations.

Collectively, our results reveal substantial intrageneric diversification within environmental *Legionella* populations inhabiting engineered water systems. The recognition of *Legionella pelazae* sp. nov. and *Legionella navarrensis* sp. nov. not only enriches the taxonomic framework of the genus but also contributes to a more comprehensive understanding of evolutionary processes operating in anthropogenic aquatic ecosystems. These findings emphasize that the genomic diversity of environmental *Legionella* remains incompletely explored and that engineered aquatic ecosystems may harbor deeply divergent lineages whose evolutionary trajectories have yet to be resolved.

## Data Availability

The datasets generated during the current study were deposited and are available at the National Center for Biotechnology Information (NCBI) public database under the flowing accession number: *Legionella pelazae* sp. nov strain CNM-1927-20^T^: sequencing project: JBNVZS000000000; Biosample: SAMN48408482; Bioproject: PRJNA1260987; *mip gene* sequence: PV698486; 16S rRNA gene sequence: PV642447. *Legionella navarrensis* sp. nov strain CNM-4043-24^T^: sequencing project: JBNVZR000000000.1; Biosample: SAMN48404767; Bioproject: PRJNA1260922; *mip gene* sequence: PV698485; 16S rRNA gene sequence: PV642446.

## References

[B1] Alcock B. P. Huynh W. Chalil R. Smith K. W. Raphenya A. R. Wlodarski M. A. (2023). CARD 2023: expanded curation, support for machine learning, and resistome prediction at the comprehensive antibiotic resistance database. Nucleic Acids Res. 51, D690–d699. doi: 10.1093/nar/gkac920 36263822 PMC9825576

[B2] Ankenbrand M. J. Keller A. (2016). bcgTree: automatized phylogenetic tree building from bacterial core genomes. Genome 59, 783–791. doi: 10.1139/gen-2015-0175 27603265

[B3] Benson R. F. Thacker W. L. Waters R. P. Quinlivan P. A. Mayberry W. R. Brenner D. J. Wilkinson H. W. (1989). Legionella quinlivanii sp. nov. isolated from water. Current Microbiol. 18, 195–197. doi: 10.1007/BF01569569

[B4] Buchfink B. Reuter K. Drost H. G. (2021). Sensitive protein alignments at tree-of-life scale using DIAMOND. Nat. Methods 18, 366–368. doi: 10.1038/s41592-021-01101-x PMC8026399 33828273

[B5] Camacho C. Coulouris G. Avavgyan V. Ma N. Papadopoulos J. Bealer K. . (2009). BLAST+: architecture and applications. BMC Bioinform. 10:421. doi: 10.1186/1471-2105-10-421 20003500 PMC2803857

[B6] Cantalapiedra C. P. Hernandez-Plaza A. Letunic I. Bork P. Huerta-Cepas J. (2021). eggNOG-mapper v2: functional annotation, orthology assignments, and domain prediction at the metagenomic scale. Mol. Biol. Evol. 38, 5825–5829. doi: 10.1093/molbev/msab293 PMC8662613 34597405

[B7] Carattoli A. Zankari E. Garcia-Fernandez A. Voldby Laesen M. Lund O. Villa L. . (2014). *In silico* detection and typing of plasmids using PlasmidFinder and plasmid multilocus sequence typing. Antimicrob. Agents Chemother. 58, 3895–3903. doi: 10.1128/AAC.02412-14 24777092 PMC4068535

[B8] Cazalet C. Rusniok C. Bruggemann H. Zidane N. Magnier A. Ma L. . (2004). Evidence in the Legionella pneumophila genome for exploitation of host cell functions and high genome plasticity. Nat. Genet. 36, 1165–1173. doi: 10.1038/ng1447 15467720

[B9] Chen L. Yang J. Yu J. Yao Z. Sun L. Shen Y. . (2005). VFDB: a reference database for bacterial virulence factors. Nucleic Acids Res. 33, D325–D328. doi: 10.1093/nar/gki008 PMC539962 15608208

[B10] Chen S. Zhou Y. Chen Y. Gu J. (2018). fastp: an ultra-fast all-in-one FASTQ preprocessor. Bioinformatics 34, i884–i890. doi: 10.1093/bioinformatics/bty560 30423086 PMC6129281

[B11] Chun J. Rainey F. A. (2014). Integrating genomics into the taxonomy and systematics of the Bacteria and Archaea. Int. J. Syst. Evol. Microbiol. 64, 316–324. doi: 10.1099/ijs.0.054171-0 24505069

[B12] Clausen P. Aarestrup F. M. Lund O. (2018). Rapid and precise alignment of raw reads against redundant databases with KMA. BMC Bioinformat. 19:307. doi: 10.1186/s12859-018-2336-6 30157759 PMC6116485

[B13] Cowan S. T. Steel K. J. Barrow G. I. Feltham R. K. A. (1993). Cowan and Steel's Manual for the Identification of Medical Bacteria. Cambridge; New York, NY: Cambridge University Press.

[B14] Eddy S. R. (2011). Accelerated profile HMM searches. PLoS Comput. Biol. 7:e1002195. doi: 10.1371/journal.pcbi.1002195 22039361 PMC3197634

[B15] Edgar R. C. (2004). MUSCLE: multiple sequence alignment with high accuracy and high throughput. Nucleic Acids Res. 32, 1792–1797. doi: 10.1093/nar/gkh340 15034147 PMC390337

[B16] Ewels P. Magnusson M. Lundin S. Kaller M. (2016). MultiQC: summarize analysis results for multiple tools and samples in a single report. Bioinformatics 32, 3047–3048. doi: 10.1093/bioinformatics/btw354 27312411 PMC5039924

[B17] Falkinham J. O. (2020). Living with legionella and other waterborne pathogens. Microorganisms 8:2026. doi: 10.3390/microorganisms8122026 PMC7766883 33352932

[B18] Feldgarden M. Brover V. Gonzalez-Escalona N. Frye J. G. Haendiges J. Haft D. H. . (2021). AMRFinderPlus and the reference gene catalog facilitate examination of the genomic links among antimicrobial resistance, stress response, and virulence. Sci. Rep. 11:12728. doi: 10.1038/s41598-021-91456-0 PMC8208984 34135355

[B19] Fields B. S. Benson R. F. Besser R. E. (2002). Legionella and Legionnaires' disease: 25 years of investigation. Clin. Microbiol. Rev. 15, 506–526. doi: 10.1128/CMR.15.3.506-526.2002 12097254 PMC118082

[B20] Fry N. K. Afshar B. Bellamy W. Underwood A. P. Ratcliff R. M. Harrison T. G. (2007). Identification of Legionella spp. by 19 European reference laboratories: results of the European Working Group for Legionella Infections External Quality Assessment Scheme using DNA sequencing of the macrophage infectivity potentiator gene and dedicated online tools. Clin. Microbiol. Infect. 13, 1119–1124. doi: 10.1111/j.1469-0691.2007.01808.x 17725649

[B21] Gerhardt P. Murray R. G. E. Wood W. A. Krieg N. R. (1994). Methods for General and Molecular Bacteriology. Washington, D.C.: American Society for Microbiology.

[B22] Gomez-Valero L. Buchrieser C. (2019). Intracellular parasitism, the driving force of evolution of Legionella pneumophila and the genus Legionella. Genes Immun. 20, 394–402. doi: 10.1038/s41435-019-0074-z 31053752

[B23] Gomez-Valero L. Rusniok C. Crason D. Mondino S. Perez-Cobas A. E. Rolando M. . (2019). More than 18,000 effectors in the Legionella genus genome provide multiple, independent combinations for replication in human cells. Proc. Natl. Acad. Sci. U S A. 116, 2265–2273. doi: 10.1073/pnas.1808016116 PMC6369783 30659146

[B24] Gomez-Valero L. Rusniok C. Jarraud S. Vacherie B. Rouy Z. Barbe V. . (2011). Extensive recombination events and horizontal gene transfer shaped the Legionella pneumophila genomes. BMC Genom. 12:536. doi: 10.1186/1471-2164-12-536 22044686 PMC3218107

[B25] Gurevich A. Saveliev V. Vyahni N. Tesler G. (2013). QUAST: quality assessment tool for genome assemblies. Bioinformatics 29, 1072–1075. doi: 10.1093/bioinformatics/btt086 23422339 PMC3624806

[B26] Hammes F. Gabrielli M. Cavallaro A. Eichelberg A. Barielli S. Bigler M. . (2025). Foresight 2035: a perspective on the next decade of research on the management of Legionella spp. in engineered aquatic environments. FEMS Microbiol. Rev. 49:22. doi: 10.1093/femsre/fuaf022 PMC12151150 40424003

[B27] Hasman H. Saputra D. Sicheritz-Ponten T. Lund O. Svendsen C. A. Frimodt-Moller N. . (2014). Rapid whole-genome sequencing for detection and characterization of microorganisms directly from clinical samples. J. Clin. Microbiol. 52, 139–146. doi: 10.1128/JCM.02452-13 24172157 PMC3911411

[B28] Huerta-Cepas J. Szklarczyk D. Heller D. Hernandez-Plaza A. Forslund S. K. Cook H. . (2019). eggNOG 5.0: a hierarchical, functionally and phylogenetically annotated orthology resource based on 5090 organisms and 2502 viruses. Nucleic Acids Res. 47, D309–d314. doi: 10.1093/nar/gky1085 PMC6324079 30418610

[B29] Hunt M. Mather A. E. Sanchez-Buso L. Page A. J. Parkhill J. Keane J. A. Harris S. R. (2017). ARIBA: rapid antimicrobial resistance genotyping directly from sequencing reads. Microb. Genom. 3:e000131. doi: 10.1099/mgen.0.000131 PMC5695208 29177089

[B30] Hunter J. D. (2007). Matplotlib: a 2D graphics environment. Comput. Sci. Eng. 9, 90–95. doi: 10.1109/MCSE.2007.55

[B31] Hyatt D. Chen G. L. Locascio P. F. Land M. L. Larimer F. W. Hauser L. J. (2010). Prodigal: prokaryotic gene recognition and translation initiation site identification. BMC Bioinformat. 11:119. doi: 10.1186/1471-2105-11-119 PMC2848648 20211023

[B32] Kim D. Song L. Breitwieser F. P. Salzberg S. L. (2016). Centrifuge: rapid and sensitive classification of metagenomic sequences. Genome Res. 26, 1721–1729. doi: 10.1101/gr.210641.116 27852649 PMC5131823

[B33] Kim M. Oh H. S. Park S. C. Chun J. (2014). Towards a taxonomic coherence between average nucleotide identity and 16S rRNA gene sequence similarity for species demarcation of prokaryotes. Int. J. Syst. Evol. Microbiol. 64, 346–351. doi: 10.1099/ijs.0.059774-0 24505072

[B34] Konstantinidis K. T. Tiedje J. M. (2005). Genomic insights that advance the species definition for prokaryotes. Proc. Natl. Acad. Sci. U S A. 102, 2567–2572. doi: 10.1073/pnas.0409727102 15701695 PMC549018

[B35] Kumar S. Stecher G. Li M. Knyaz C. Tamura K. (2018). MEGA X: molecular evolutionary genetics analysis across computing platforms. Mol. Biol. Evol. 35, 1547–1549. doi: 10.1093/molbev/msy096 29722887 PMC5967553

[B36] Larsen M. V. Cosentino S. Lukjancenko O. Saputra D. Rasmussen S. Hasman H. . (2014). Benchmarking of methods for genomic taxonomy. J. Clin. Microbiol. 52, 1529–1539. doi: 10.1128/JCM.02981-13 PMC3993634 24574292

[B37] Le S. Q. Gascuel O. (2008). An improved general amino acid replacement matrix. Mol. Biol. Evol. 25, 1307–1320. doi: 10.1093/molbev/msn067 18367465

[B38] Lo Presti F. Riffard S. Meugnier H. Reyrolle M. Lasne Y. Grimont P. A. . (2001). Legionella gresilensis sp. nov. and Legionella beliardensis sp. nov., isolated from water in France. Int. J. Syst. Evol. Microbiol. 51, 1949–1957. doi: 10.1099/00207713-51-6-1949 11760933

[B39] Lu J. Breitwieser F. P. Thielen P. Salzberg S. L. (2017). Bracken: estimating species abundance in metagenomics data. PeerJ. Comput. Sci. 3:104. doi: 10.7717/peerj-cs.104 PMC12016282 40271438

[B40] McKinner W. (2010). “Data Structures for Statistical Computing in Python,” in Proceedings of the 9th Python in Science Conference, 51–56. doi: 10.25080/Majora-92bf1922-00a

[B41] Meier-Kolthoff J. P. Auch A. F. Klenk H. P. Goker M. (2013). Genome sequence-based species delimitation with confidence intervals and improved distance functions. BMC Bioinformat. 14:60. doi: 10.1186/1471-2105-14-60 PMC3665452 23432962

[B42] Meier-Kolthoff J. P. Carbasse J. S. Peinado-Olarte R. L. Goker M. (2022). TYGS and LPSN: a database tandem for fast and reliable genome-based classification and nomenclature of prokaryotes. Nucleic Acids Res. 50, D801–d807. doi: 10.1093/nar/gkab902 34634793 PMC8728197

[B43] Meier-Kolthoff J. P. Göker M. (2019). TYGS is an automated high-throughput platform for state-of-the-art genome-based taxonomy. Nat. Commun. 10:2182. doi: 10.1038/s41467-019-10210-3 31097708 PMC6522516

[B44] Menzel P. Ng K. L. Krogh A. (2016). Fast and sensitive taxonomic classification for metagenomics with Kaiju. Nat. Commun. 7:11257. doi: 10.1038/ncomms11257 PMC4833860 27071849

[B45] MIDI. (2008). Sherlock Microbial Identification System Operating Manual, version 6.1. DE: MIDI, Inc Newark.

[B46] Minh B. Q. Schmidt H. A. Chernomor O. Schrempf D. Woodhams M. D. Von Haeseler A. . (2020). IQ-TREE 2: new models and efficient methods for phylogenetic inference in the genomic era. Mol. Biol. Evol. 37, 1530–1534. doi: 10.1093/molbev/msaa015 PMC7182206 32011700

[B47] Monzon S. Sola J. P. Julia M. (2021). PlasmidID, 1.6.5 version. Available online at: https://github.com/BU-ISCIII/plasmidID (Accessed April 2026)

[B48] Murray C. S. Gao Y. Wu M. (2021). Re-evaluating the evidence for a universal genetic boundary among microbial species. Nat. Commun. 12:4059. doi: 10.1038/s41467-021-24128-2 PMC8263626 34234129

[B49] Newton H. J. Hartland E. L. Machner M. P. (2018). Editorial: biology and pathogenesis of legionella. Front. Cell Infect. Microbiol. 8:328. doi: 10.3389/fcimb.2018.00328 30283746 PMC6156375

[B50] Nisar M. A. Ross K. E. Brown M. H. Bentham R. Whiley H. (2020). Water stagnation and flow obstruction reduces the quality of potable water and increases the risk of legionelloses. Front. Environ. Sci. 8:1611. doi: 10.3389/fenvs.2020.611611

[B51] Oliva G. Sahr T. Buchrieser C. (2018). The life cycle of L. pneumophila: cellular differentiation is linked to virulence and metabolism. Front. Cell Infect. Microbiol. 8:3. doi: 10.3389/fcimb.2018.00003 PMC5780407 29404281

[B52] Park M. Y. Ko K. S. Lee H. K. Park M. S. Kook Y. H. (2003). Legionella busanensis sp. nov., isolated from cooling tower water in Korea. Int. J. Syst. Evol. Microbiol. 53, 77–80. doi: 10.1099/ijs.0.02268-0 12656155

[B53] Parks D. H. Imelfort M. Skennerton C. T. Hugenholtz P. Tyson G. W. (2015). CheckM: assessing the quality of microbial genomes recovered from isolates, single cells, and metagenomes. Genome Res. 25, 1043–1055. doi: 10.1101/gr.186072.114 25977477 PMC4484387

[B54] Price C. T. D. Hanford H. E. Al-Quadan T. Santic M. Shin C. J. Da'as M. S. J. . (2024). Amoebae as training grounds for microbial pathogens. mBio. 15:e0082724. doi: 10.1128/mbio.00827-24 38975782 PMC11323580

[B55] Python Software Foundation. Python Language Reference version 3.9. Available online at: https://www.python.org/ (Accessed April 23, 2026).

[B56] Ratcliff R. M. Lanser J. A. Manning P. A. Heuzenroeder M. W. (1998). Sequence-based classification scheme for the genus Legionella targeting the mip gene. J. Clin. Microbiol. 36, 1560–1567. doi: 10.1128/JCM.36.6.1560-1567.1998 9620377 PMC104877

[B57] Richards A. M. Von Dwingelo J. E. Price C. T. Abu Kwaik Y. (2013). Cellular microbiology and molecular ecology of Legionella-amoeba interaction. Virulence 4, 307–314. doi: 10.4161/viru.24290 PMC3710333 23535283

[B58] Richter M. Rosselló-Móra R. (2009). Shifting the genomic gold standard for the prokaryotic species definition. Proc. Natl. Acad. Sci. U S A. 106, 19126–19131. doi: 10.1073/pnas.0906412106 PMC2776425 19855009

[B59] Rodriguez R. L. Jain C. Conrad R. E. Aluru S. Konstantinidis K. T. (2021). Reply to: “Re-evaluating the evidence for a universal genetic boundary among microbial species”. Nat. Commun. 12:4060. doi: 10.1038/s41467-021-24129-1 34234115 PMC8263725

[B60] Sasser M. (1990). Identification of Bacteria by Gas Chromatography of Cellular Fatty Acids. MIDI technical note 101. Newark, DE: MIDI inc.

[B61] Seemann T. (2014). Prokka: rapid prokaryotic genome annotation. Bioinformatics 30, 2068–2069. doi: 10.1093/bioinformatics/btu153 24642063

[B62] Sewell M. Farley C. Portal E. A. R. Lindsay D. Ricci M. L. Jarraud S. . (2025). Broth microdilution protocol for determining antimicrobial susceptibility of Legionella pneumophila to clinically relevant antimicrobials. J. Microbiol. Methods 228:107071. doi: 10.1016/j.mimet.2024.107071 39706371

[B63] Silva M. Machado M. P. Silva D. N. Rossi M. Moran-Gilad J. Santos S. . (2018). chewBBACA: a complete suite for gene-by-gene schema creation and strain identification. Microb. Genom. 4:166. doi: 10.1099/mgen.0.000166 29543149 PMC5885018

[B64] Stamatakis A. (2006). RAxML-VI-HPC: maximum likelihood-based phylogenetic analyses with thousands of taxa and mixed models. Bioinformatics 22, 2688–2690. doi: 10.1093/bioinformatics/btl446 16928733

[B65] Stamouli S. Beber M. E. Normark T. Christensen T. A. Andersson-Li L. Borry M. . (2023). nf-core/taxprofiler: highly parallelised and flexible pipeline for metagenomic taxonomic classification and profiling. bioRxiv 2023:3221. doi: 10.1101/2023.10.20.563221

[B66] Talavera G. Castresana J. (2007). Improvement of phylogenies after removing divergent and ambiguously aligned blocks from protein sequence alignments. Syst. Biol. 56, 564–577. doi: 10.1080/10635150701472164 17654362

[B67] Tamura K. Nei M. (1993). Estimation of the number of nucleotide substitutions in the control region of mitochondrial DNA in humans and chimpanzees. Mol. Biol. Evol. 10, 512–526. 8336541 10.1093/oxfordjournals.molbev.a040023

[B68] Tamura K. Stecher G. Kumar S. (2021). MEGA11: molecular evolutionary genetics analysis version 11. Mol. Biol. Evol. 38, 3022–3027. doi: 10.1093/molbev/msab120 33892491 PMC8233496

[B69] Vm D. Peltzer A. Straub D. Bot N.-C. Wuennemann F. Garcia M. U. . (2024). nf-core/bacass: v2.4.0. Zenodo. Available online at: https://zenodo.org/records/14180424

[B70] Wick R. R. Judd L. M. Gorrie C. L. Holt K. E. (2017). Unicycler: resolving bacterial genome assemblies from short and long sequencing reads. PLoS Comput. Biol. 13:e1005595. doi: 10.1371/journal.pcbi.1005595 28594827 PMC5481147

[B71] Wilkinson H. W. Thacker W. L. Benson R. F. Polt S. S. Brookings E. Mayberry W. R. . (1987). Legionella birminghamensis sp. nov. isolated from a cardiac transplant recipient. J. Clin. Microbiol. 25, 2120–2122. doi: 10.1128/jcm.25.11.2120-2122.1987 3320081 PMC269423

[B72] Wood D. E. Lu J. Langmead B. (2019). Improved metagenomic analysis with Kraken 2. Genome Biol. 20:257. doi: 10.1186/s13059-019-1891-0 31779668 PMC6883579

[B73] Yao X. H. Shen F. Hao J. Huang L. Keng B. (2024). A review of Legionella transmission risk in built environments: sources, regulations, sampling, and detection. Front. Public Health 12:1415157. doi: 10.3389/fpubh.2024.1415157 PMC11309999 39131570

[B74] Yoon S. H. Ha S. M. Lim J. Kwon S. Chun J. (2017). A large-scale evaluation of algorithms to calculate average nucleotide identity. Antonie Van Leeuwenhoek 110, 1281–1286. doi: 10.1007/s10482-017-0844-4 28204908

