# Supplementary material for: Legionella pelazae sp. nov. and Legionella navarrensis sp. nov., two novel Legionella species, reveal deep genomic diversification within engineered water systems
Source: Front Microbiol. 2026 Sep 9;17:1828313. doi: 10.3389/fmicb.2026.1828313 (PMC13599621; doi:10.3389/fmicb.2026.1828313)
Supplement: Supplementary file 1 [file Supplementary_file_1.docx]

Supplementary Material

## 1.1 Supplementary Tables

**Supplementary Table S1**. Similarity results of genes sequence comparisons among CNM-1927-20^T^ (PV642447) and CNM-4043-24^T^ (PV642446) and other *Legionella* strains for 16S rRNA gene. The accession numbers are indicated in parentheses.

|  | **CNM-1927-20^T^**  **(1,431 bp)** | | | | **CNM-4043-24^T^**  **(1,429 bp)** | | | | | | |
| --- | --- | --- | --- | --- | --- | --- | --- | --- | --- | --- | --- |
| **Species (GenBank AN)** | **similarity (%)** | **Coverlength (%)** | | | | **similarity (%)** | **Coverlength (%)** | | | |  |
| CNM-4043-24^T^ (PV642446) | 94.13 | | 100 |  | | | |  |  |  |  |
| *L. adelaidensis* (NR_044952.1) | 94.57 | | 98 | 94.05 | | | | 98 | |  |  |
| *L. anisa* (NR_036988.1) | 93.98 | | 96 | 94.05 | | | | 96 | |  |  |
| *L. antarctica* (AP022839) | 94.96 | | 98 | 94.88 | | | | 98 | |  |  |
| *L. beliardensis* (NR_024968.1) | 96.74 | | 98 | 93.61 | | | | 98 | |  |  |
| *L. birminghamensis* (NR_044953.1) | 95.47 | | 97 | 94.32 | | | | 97 | |  |  |
| *L. bononiensis* (OK495709.1) | 94.14 | | 98 | 93.78 | | | | 97 | |  |  |
| *L. bozemanae* (NR_125584.1) | 94.87 | | 37 | 93.73 | | | | 36 | |  |  |
| *L. brunensis* (NR_026520.1) | 94.45 | | 98 | 93.67 | | | | 98 | |  |  |
| *L. busanensis* (NR_025196.1) | 97.93 | | 98 | 94.28 | | | | 97 | |  |  |
| *L. cardiaca* (NR_109426.1) | 94.38 | | 96 | 93.65 | | | | 95 | |  |  |
| *L. cherrii* (NR_044954.1) | 93.09 | | 93 | 94.01 | | | | 93 | |  |  |
| *L. cincinnatiensis* (NR_044955.1) | 94.10 | | 97 | 93.61 | | | | 97 | |  |  |
| *L. drancourtii* (NR_026335.1) | 93.71 | | 94 | 94.45 | | | | 94 | |  |  |
| *L. dresdenensis* (NR_115062.1) | 95.89 | | 99 | 94.96 | | | | 98 | |  |  |
| *L. drozanskii* (NR_036803.1) | 94.67 | | 98 | 94.52 | | | | 98 | |  |  |
| *L. dumoffii* (NR_119025.1) | 93.08 | | 98 | 93.65 | | | | 97 | |  |  |
| *L. erythra* (NR_029369.1) | 94.71 | | 98 | 94.92 | | | | 97 | |  |  |
| *L. fairfieldensis* (NR_044956.1) | 94.20 | | 98 | 93.48 | | | | 98 | |  |  |
| *L. fallonii* (NR_036805.1) | 94.33 | | 98 | 94.46 | | | | 98 | |  |  |
| *L. feeleii* (NR_026117.1) | 93.30 | | 98 | 94.15 | | | | 97 | |  |  |
| *L. geestiana* (NR_044957.1) | 91.81 | | 97 | 94.54 | | | | 97 | |  |  |
| *L. gormanii* (NR_026521.1) | 93.82 | | 98 | 94.25 | | | | 98 | |  |  |
| *L. gratiana* (NR_044958.1) | 93.75 | | 96 | 94.35 | | | | 96 | |  |  |
| *L. gresilensis* (NR_028744.1) | 97.21 | | 97 | 94.21 | | | | 97 | |  |  |
| *L. hackeliae* (NR_104894.1) | 94.79 | | 98 | 93.65 | | | | 97 | |  |  |
| *L. impletisoli* (NR_041321.1) | 95.18 | | 99 | 93.83 | | | | 98 | |  |  |
| *L. israelensis* (NR_036894.1) | 93.21 | | 98 | 91.92 | | | | 97 | |  |  |
| *L. jamestowniensis* (NR_044959.1) | 93.80 | | 97 | 92.80 | | | | 96 | |  |  |
| *L. jordanis* (NR_036992.1) | 94.39 | | 98 | 93.68 | | | | 98 | |  |  |
| *L. lansingensis* (NR_044960.1) | 92.73 | | 97 | 92.30 | | | | 96 | |  |  |
| *L. londiniensis* (NR_044963.1) | 95.05 | | 97 | 93.31 | | | | 97 | |  |  |
| *L. longbeachae* (NR_042906.1) | 94.17 | | 97 | 94.03 | | | | 97 | |  |  |
| *L. lytica* (NR_026334.1) | 93.73 | | 98 | 94.73 | | | | 98 | |  |  |
| *L. maceachernii* (NR_041790.1) | 95.65 | | 98 | 93.80 | | | | 97 | |  |  |
| *L. maioricensis* (OL472331.1) | 94.31 | | 98 | 94.74 | | | | 98 | |  |  |
| *L. massiliensis* (NR_109415.1) | 94.89 | | 98 | 94.31 | | | | 98 | |  |  |
| *L. micdadei* (NR_041791.1) | 95.43 | | 98 | 93.64 | | | | 97 | |  |  |
| *L. moravica* (NR_044962.1) | 93.00 | | 97 | 92.79 | | | | 96 | |  |  |
| *L. nagasakiensis* (NR_116420.1) | 95.01 | | 98 | 93.30 | | | | 97 | |  |  |
| *L. nautarum* (NR_044961.1) | 93.63 | | 96 | 93.33 | | | | 96 | |  |  |
| *L. norrlandica* (KJ796839.1) | 95.10 | | 98 | 94.17 | | | | 98 | |  |  |
| *L. oakridgensis* (NR_036990.1) | 94.35 | | 97 | 92.54 | | | | 96 | |  |  |
| *L. parisiensis* (NR_044964.1) | 94.19 | | 96 | 94.48 | | | | 96 | |  |  |
| *L. petroniana* (PV189399.1) | 94.60 | | 91 | 95.67 | | | | 91 | |  |  |
| *L. pneumophila* strain *Philadelphia*1 (NR_074231.1) | 94.89 | | 98 | 94.46 | | | | 98 | |  |  |
| *L. pneumophila* subsp. *fraseri* (NR_104921.1) | 94.41 | | 98 | 94.47 | | | | 98 | |  |  |
| *L. pneumophila* subsp.*pascullei* (NR_041742.1) | 94.54 | | 98 | 94.60 | | | | 98 | |  |  |
| *L. qingyii* (NR_171519.1) | 94.24 | | 98 | 94.74 | | | | 98 | |  |  |
| *L. quateirensis* (NR_044965.1) | 93.31 | | 96 | 92.88 | | | | 96 | |  |  |
| *L. quinlivanii* (NR_044966.1) | 94.39 | | 97 | 93.39 | | | | 97 | |  |  |
| *L. resiliens* (OL804581.1) | 94.11 | | 98 | 94.6 | | | | 98 | |  |  |
| *L. rowbothamii* (NR_036804.1) | 94.08 | | 98 | 95.01 | | | | 98 | |  |  |
| *L. rubrilucens* (NR_036991.1) | 94.96 | | 98 | 95.18 | | | | 98 | |  |  |
| *L. sainthelensi* (NR_044967.1) | 93.50 | | 96 | 93.37 | | | | 96 | |  |  |
| *L. santicrucis* (NR_117698.1) | 93.99 | | 91 | 94.31 | | | | 91 | |  |  |
| *L. saoudiensis* (NR_152697.1) | 94.31 | | 98 | 94.89 | | | | 98 | |  |  |
| *L. septentrionalis* (NR_180074.1) | 94.18 | | 98 | 93.25 | | | | 98 | |  |  |
| *L. shakespearei* (NR_119320.1) | 93.52 | | 97 | 93.43 | | | | 97 | |  |  |
| *L. spiritensis* (NR_117699.1) | 95.00 | | 97 | 94.22 | | | | 97 | |  |  |
| *L. steelei* (NR_117981.1) | 93.91 | | 95 | 94.57 | | | | 95 | |  |  |
| *L. steigerwaltii* (NR_044969.1) | 93.44 | | 96 | 93.80 | | | | 96 | |  |  |
| *L. taurinensis* (NR_043927.1) | 95.38 | | 98 | 95.24 | | | | 98 | |  |  |
| *L. thermalis* (NR_146358.1) | 94.00 | | 97 | 92.65 | | | | 97 | |  |  |
| *L. tucsonensis* (NR_026522.1) | 93.92 | | 96 | 93.85 | | | | 96 | |  |  |
| *L. tunisiensis* (NR_109416.1) | 94.82 | | 98 | 94.82 | | | | 98 | |  |  |
| *L. wadsworthii* (NR_044970.1) | 93.83 | | 97 | 94.48 | | | | 97 | |  |  |
| *L. waltersii* (NR_024969.1) | 94.67 | | 98 | 94.75 | | | | 98 | |  |  |
| *L. worsleiensis* (NR_044971.1) | 94.06 | | 97 | 93.83 | | | | 97 | |  |  |
| *L. yabuuchiae* (NR_041322.1) | 95.12 | | 94 | 94.22 | | | |  | |  |  |

**Supplementary Table S2**. Similarity results of genes sequence comparisons among CNM-1927-20^T^ (PV698486), CNM-4043-24^T^ (PV698485) and other *Legionella* strains for *mip* gen. The accession numbers are indicated in parentheses.

|  | **CNM-1927-20^T^**  **(697 bp)** | | | | **CNM-4043-24^T^**  **(619 bp)** | | | | | | |  |
| --- | --- | --- | --- | --- | --- | --- | --- | --- | --- | --- | --- | --- |
| **Species (GenBank AN)** | **similarity**  **(%)** | | | **Coverlength (%)** | | | **similarity**  **(%)** | | **Coverlength (%)** | | | |
| CNM-4043-24^T^ (PV642446) | | 77.07 | 74 | | |  | |  | | |  |  |
| *L. adelaidensis* (NR_044952.1) | | 72.66 | 83 | | | 69.63 | | | | 100 | |  |
| *L. anisa* (NR_036988.1) | | 75.68 | 78 | | | 71.72 | | | | 87 | |  |
| *L. antarctica* (AP022839) | | 75.09 | 76 | | | 71.91 | | | | 100 | |  |
| *L. beliardensis* (NR_024968.1) | | 81.92 | 98 | | | 74.91 | | | | 88 | |  |
| *L. birminghamensis* (NR_044953.1) | | 76.35 | 87 | | | 73.98 | | | | 87 | |  |
| *L. bononiensis* (OK495709.1) | | 75.09 | 77 | | | 72.61 | | | | 87 | |  |
| *L. bozemanae* (NR_125584.1) | | 75.23 | 76 | | | 70.88 | | | | 87 | |  |
| *L. brunensis* (NR_026520.1) | | 76.94 | 81 | | | 73.88 | | | | 90 | |  |
| *L. busanensis* (NR_025196.1) | | 90.02 | 58 | | | 74.63 | | | | 65 | |  |
| *L. cardiaca* (NR_109426.1) | | 78.18 | 75 | | | 72.21 | | | | 79 | |  |
| *L. cherrii* (NR_044954.1) | | 76.55 | 76 | | | 72.21 | | | | 99 | |  |
| *L. cincinnatiensis* (NR_044955.1) | | 74.91 | 77 | | | 71.89 | | | | 99 | |  |
| *L. drancourtii* (NR_026335.1) | | 76.54 | 77 | | | 71.40 | | | | 88 | |  |
| *L. dresdenensis* (NR_115062.1) | | 76.91 | 73 | | | 73.98 | | | | 83 | |  |
| *L. drozanskii* (NR_036803.1) | | 77.57 | 77 | | | 70.90 | | | | 87 | |  |
| *L. dumoffii* (NR_119025.1) | | 76.34 | 72 | | | 72.49 | | | | 85 | |  |
| *L. erythra* (NR_029369.1) | | 75.66 | 77 | | | 76.40 | | | | 86 | |  |
| *L. fairfieldensis* (NR_044956.1) | | 74.23 | 79 | | | 70.23 | | | | 99 | |  |
| *L. fallonii* (NR_036805.1) | | 76.89 | 76 | | | 72.09 | | | | 97 | |  |
| *L. feeleii* (NR_026117.1) | | 79.60 | 79 | | | 73.14 | | | | 100 | |  |
| *L. geestiana* (NR_044957.1) | | 68.66 | 58 | | | 67.65 | | | | 65 | |  |
| *L. gormanii* (NR_026521.1) | | 76.59 | 99 | | | 70.73 | | | | 98 | |  |
| *L. gratiana* (NR_044958.1) | | 74.05 | 72 | | | 71.77 | | | | 99 | |  |
| *L. gresilensis* (NR_028744.1) | | 91.51 | 98 | | | 76.16 | | | | 83 | |  |
| *L. hackeliae* (NR_104894.1) | | 80.38 | 76 | | | 76.81 | | | | 87 | |  |
| *L. impletisoli* (NR_041321.1) | | 74.91 | 39 | | | 66.91 | | | | 66 | |  |
| *L. israelensis* (NR_036894.1) | | 74.76 | 72 | | | 69.43 | | | | 99 | |  |
| *L. jamestowniensis* (NR_044959.1) | | 80.87 | 73 | | | 74.63 | | | | 87 | |  |
| *L. jordanis* (NR_036992.1) | | 75.84 | 77 | | | 72.20 | | | | 87 | |  |
| *L. lansingensis* (NR_044960.1) | | 77.93 | 77 | | | 72.57 | | | | 91 | |  |
| *L. londiniensis* (NR_044963.1) | | 73.98 | 42 | | | 69.83 | | | | 67 | |  |
| *L. longbeachae* (NR_042906.1) | | 74.81 | 76 | | | 72.50 | | | | 96 | |  |
| *L. lytica* (NR_026334.1) | | 76.88 | 74 | | | 71.79 | | | | 87 | |  |
| *L. maceachernii* (NR_041790.1) | | 77.71 | 75 | | | 73.26 | | | | 88 | |  |
| *L. maioricensis* (OL472331.1) | | 77.27 | 73 | | | 71.29 | | | | 100 | |  |
| *L. massiliensis* (NR_109415.1) | | 75.94 | 73 | | | 73.52 | | | | 87 | |  |
| *L. micdadei* (NR_041791.1) | | 76.46 | 69 | | | 71.30 | | | | 89 | |  |
| *L. moravica* (NR_044962.1) | | 74.24 | 76 | | | 72.66 | | | | 87 | |  |
| *L. nagasakiensis* (NR_116420.1) | | 71.95 | 67 | | | 68.99 | | | | 97 | |  |
| *L. nautarum* (NR_044961.1) | | 77.32 | 76 | | | 71.24 | | | | 87 | |  |
| *L. norrlandica* (KJ796839.1) | | 74.95 | 73 | | | 71.15 | | | | 99 | |  |
| *L. oakridgensis* (NR_036990.1) | | 73.23 | 67 | | | 70.83 | | | | 100 | |  |
| *L. parisiensis* (NR_044964.1) | | 74.90 | 72 | | | 70.11 | | | | 100 | |  |
| *L. petroniana* (PV189399.1) | | 79.06 | 78 | | | 74.33 | | | | 78 | |  |
| *L. pneumophila* strain *Philadelphia*1 (NR_074231.1) | | 74.34 | 77 | | | 70.19 | | | | 100 | |  |
| *L. pneumophila* subsp. *fraseri* (NR_104921.1) | | 74.21 | 55 | | | 68.99 | | | | 65 | |  |
| *L. pneumophila* subsp.*pascullei* (NR_041742.1) | | 74.54 | 55 | | | 68.32 | | | | 65 | |  |
| *L. qingyii* (NR_171519.1) | | 76.65 | 72 | | | 73.38 | | | | 87 | |  |
| *L. quateirensis* (NR_044965.1) | | 75.05 | 78 | | | 71.93 | | | | 87 | |  |
| *L. quinlivanii* (NR_044966.1) | | 79.28 | 72 | | | 73.28 | | | | 87 | |  |
| *L. resiliens* (OL804581.1) | | 75.50 | 72 | | | 71.19 | | | | 87 | |  |
| *L. rowbothamii* (NR_036804.1) | | 78.07 | 71 | | | 71.82 | | | | 87 | |  |
| *L. rubrilucens* (NR_036991.1) | | 76.12 | 77 | | | 75.32 | | | | 87 | |  |
| *L. sainthelensi* (NR_044967.1) | | 75.76 | 76 | | | 72.30 | | | | 99 | |  |
| *L. santicrucis* (NR_117698.1) | | 75.28 | 76 | | | 71.59 | | | | 99 | |  |
| *L. saoudiensis* (NR_152697.1) | | 73.97 | 73 | | | 70.74 | | | | 87 | |  |
| *L. septentrionalis* (NR_180074.1) | | 76.17 | 76 | | | 72.41 | | | | 87 | |  |
| *L. shakespearei* (NR_119320.1) | | 75.14 | 79 | | | 72.73 | | | | 100 | |  |
| *L. spiritensis* (NR_117699.1) | | 75.76 | 76 | | | 72.46 | | | | 87 | |  |
| *L. steelei* (NR_117981.1) | | 76.97 | 76 | | | 71.75 | | | | 99 | |  |
| *L. steigerwaltii* (NR_044969.1) | | 75.43 | 76 | | | 70.39 | | | | 99 | |  |
| *L. taurinensis* (NR_043927.1) | | 76.59 | 79 | | | 75.27 | | | | 91 | |  |
| *L. thermalis* (NR_146358.1) | | 75.47 | 76 | | | 70.40 | | | | 100 | |  |
| *L. tucsonensis* (NR_026522.1) | | 78.20 | 80 | | | 73.09 | | | | 100 | |  |
| *L. tunisiensis* (NR_109416.1) | | 75.99 | 72 | | | 71.38 | | | | 87 | |  |
| *L. wadsworthii* (NR_044970.1) | | 74.45 | 78 | | | 72.19 | | | | 87 | |  |
| *L. waltersii* (NR_024969.1) | | 73.24 | 79 | | | 68.54 | | | | 100 | |  |
| *L. worsleiensis* (NR_044971.1) | | 72.51 | 54 | | | 68.24 | | | | 61 | |  |

**Supplementary Table S3.** Overall Genome Related Index (OGRI).

OrthoANIu values between genomes of strains CNM-1927-20^T^ (query strain) (A), CNM-4043-24^T^ (query strain) (B), and the closely related species of the genus *Legionella* belonging in the same clade. Digital DNA-DNA hybridization (dDDH) pairwise comparisons between genomes of strains CNM-1927-20^T^ (C), CNM-4043-24^T^ (D), and the closely related species of the genus *Legionella* belonging in the same clade. Coverage in ANI analysis and G+C content differences in dDDH analysis were expressed as percentage. (**E),** ANI values between the proposed novel strains and all validly described *Legionella* species showing ≥95 % 16S rRNA gene sequence similarity. Data are expressed as percentage. Dashes indicate comparisons not performed because the species showed <95 % 16S rRNA gene sequence similarity with that strain.

| **(A)** | **AVERAGE NUCLEOTIDE IDENTITY (OrthoANIu) STRAIN CNM-1927-20^T^** | | | | | | | | | | |  |
| --- | --- | --- | --- | --- | --- | --- | --- | --- | --- | --- | --- | --- |
|  | **Strain (accesion number)** | **Genome size (kb)** | | **ANI value (%)** | **AVERAGE ALIGNED NUCLEOTIDE (bp)** | **COVERAGE** | | | | |  |  |
|  |  |  |  |  |  | **Query strain** | | | | **Related strain** |  |  |
|  | *L. gresilensis* (GCA_900639865.1) | 3,840 | | 85.01 | 1,706,583 | 51.28 | | | | 44.44 |  |  |
|  | *L. busanensis* (UGOD01000001.1) | 3,413 | | 84.41 | 1,609,284 | 48.35 | | | | 47.14 |  |  |
|  | *L. beliardensis* (UGNV01000001.1) | 3,170 | | 76.91 | 1,269,002 | 38.13 | | | | 40.03 |  |  |
|  | *L. birminghamensis* (NZ_UGNW01000001.1) | 3,584 | | 68.98 | 658,726 | 19.79 | | | | 18.38 |  |  |
|  | *L. dresdenensis* (GCA_042649825.1) | 2,901 | | 69.57 | 626,054 | 18.81 | | | | 21.57 |  |  |
|  | *L. quinlivanii* (GCA_001467975.1) | 3,532 | | 68.98 | 665,008 | 19.98 | | | | 18.83 |  |  |
|  | CNM-4043-24^T^ ([GCA_052527395.1](https://www.ncbi.nlm.nih.gov/datasets/genome/GCA_052527395.1/)) | 2,993 | | 68.83 | 485,757 | 14.59 | | | | 16.23 |  |  |
|  |  |  | |  |  |  | | | |  |  |  |
| **(B)** | **AVERAGE NUCLEOTIDE IDENTITY (OrthoANIu) STRAIN CNM-4043-24^T^** | | | | | | | | | | | |
|  | **Strain (accesion number)** | **Genome size (kb)** | **ANI value (%)** | | **AVERAGE ALIGNED NUCLEOTIDE (bp)** | | **COVERAGE** | | | | | |
|  |  |  |  |  |  |  | **Query strain** | | **Related strain** | | | |
|  | *L. gresilensis* (GCA_900639865.1) | 3,840 | 68.87 | | 444,745 | | 14.86 | 11.58 | | | | |
|  | *L. busanensis* (UGOD01000001.1) | 3,413 | 68.86 | | 444,056 | | 14.83 | 13.01 | | | | |
|  | *L. beliardensis* (UGNV01000001.1) | 3,170 | 68.79 | | 542,354 | | 18.12 | 17.11 | | | | |
|  | *L. birminghamensis* (NZ_UGNW01000001.1) | 3,584 | 69.58 | | 623,167 | | 20.82 | 17.39 | | | | |
|  | *L. dresdenensis* (GCA_042649825.1) | 2,901 | 70.37 | | 617,208 | | 20.62 | 21.27 | | | | |
|  | *L. quinlivanii* (GCA_001467975.1) | 3,532 | 69.24 | | 659,656 | | 22.03 | 18.68 | | | | |
|  | CNM-1927-20^T^ ([GCA_052527415.1](https://www.ncbi.nlm.nih.gov/datasets/genome/GCA_052527415.1/)) | *3,397* | 68.83 | | 485,757 | | 16.23 | 14.59 | | | | |

| **(C)** | | **DIGITAL DNA-DNA HIBRIDATION (dDDH) CNM-1927-20^T^** | | | | | | | | | | | | | | | | | | | | | | | | | | | | | | | | | |
| --- | --- | --- | --- | --- | --- | --- | --- | --- | --- | --- | --- | --- | --- | --- | --- | --- | --- | --- | --- | --- | --- | --- | --- | --- | --- | --- | --- | --- | --- | --- | --- | --- | --- | --- | --- |
|  | | **Subject strain (accession number)** | **dDDH 0,** | | | | **[95 % CI]** | | | | **dDDH 4,** | | | | **[95 % CI]** | | | | **dDDH 6,** | | | | | **[95 % CI]** | | | | | **G+C Content differences** | | | | |  |  |
|  | | *L. gresilensis* (GCA_900639865.1) | 40.4 | | | | [37-43.9] | | | | 30.1 | | | | [27.7 - 32.6] | | | | 37.1 | | | | | [34.2 - 40.2] | | | | | 0.52 | | | | |  |  |
|  | | *L. busanensis* (UGOD01000001.1) | 39.3 | | | | [35.9-42.7] | | | | 28.9 | | | | [26.6 - 31.4] | | | | 35.9 | | | | | [32.9 - 38.9] | | | | | 0.71 | | | | |  |  |
|  | | *L. beliardensis* (UGNV01000001.1) | 17.6 | | | | [14.5- 21.1] | | | | 21.3 | | | | [19 - 23.7] | | | | 17.4 | | | | | [14.8 - 20.3] | | | | | 1.81 | | | | |  |  |
|  | | *L. birminghamensis* (NZ_UGNW01000001.1) | 12.9 | | | | [10.2-16.2] | | | | 19.4 | | | | [17.2 - 21.8] | | | | 13.3 | | | | | [10.9 - 16] | | | | | 8.12 | | | | |  |  |
|  | | *L. dresdenensis* (GCA_042649825.1) | 12.8 | | | | [10.1-16] | | | | 19.1 | | | | [16.9 - 21.5] | | | | 13.2 | | | | | [10.8 - 15.9] | | | | | 7.51 | | | | |  |  |
|  | | *L. quinlivanii* (GCA_001467975.1) | 12.8 | | | | [10.1-16] | | | | 19.2 | | | | [17 - 21.6] | | | | 13.1 | | | | | [10.8 - 15.9] | | | | | 7.45 | | | | |  |  |
|  | | CNM-4043-24^T^([GCA_052527395.1](https://www.ncbi.nlm.nih.gov/datasets/genome/GCA_052527395.1/)) | 12.8 | | | | [10.1 - 16.1] | | | | 19.5 | | | | [17.3 - 21.9] | | | | 13.2 | | | | | [10.8 - 16%] | | | | | 13.20 | | | | |  |  |
|  | |  | | |  | | | |  | | | |  | | | |  | | | |  | | | | |  | | | |  | | | | | |
|  | **(D)** | **DIGITAL DNA-DNA HIBRIDATION (dDDH) CNM-4043-24^T^** | | | | | | | | | | | | | | | | | | | | | | | | | | | | | | | | |  |
|  |  | **Subject strain (accession number)** | | **dDDH 0,** | | | | **[95 % CI]** | | | | **dDDH 4,** | | | | **[95 % CI]** | | | | **dDDH 6,** | | | | | **[95 % CI]** | | | | **G+C Content differences** | | | | |  |  |
|  |  | *L. gresilensis* (GCA_900639865.1) | 12.7 | | | [10.1 - 16] | | | | 18.8 | | | | [16.7 - 21.2] | | | | 13.1 | | | | | [10.8 - 15.9] | | | | | 13.72 | | | |  |  |  |  |
|  |  | *L. busanensis* (UGOD01000001.1) | 12.9 | | | [10.2 - 16.2] | | | | 19.5 | | | | [17.3 - 21.9] | | | | 13.3 | | | | | [10.9 - 16] | | | | | 13.91 | | | |  |  |  |  |
|  |  | *L. beliardensis* (UGNV01000001.1) | 12.9 | | | [10.2 - 16.2] | | | | 19.2 | | | | [17 - 21.6] | | | | 13.3 | | | | | [10.9 - 16] | | | | | 11.39 | | | |  |  |  |  |
|  |  | *L. birminghamensis* (NZ_UGNW01000001.1) | 13 | | | [10.3 - 16.3] | | | | 19.6 | | | | [17.4 - 22] | | | | 13.3 | | | | | [11 - 16.1] | | | | | 5.08 | | | |  |  |  |  |
|  |  | *L. dresdenensis* (GCA_042649825.1) | 13.2 | | | [10.4 - 16.5] | | | | 18.4 | | | | [16.3 - 20.8] | | | | 13.5 | | | | | [11.1 - 16.3] | | | | | 5.70 | | | |  |  |  |  |
|  |  | *L. quinlivanii* (GCA_001467975.1) | 12.9 | | | [10.2 - 16.2] | | | | 18.6 | | | | [16.4 - 21] | | | | 13.3 | | | | [10.9 - 16.1] | | | | | 5.75 | | | |  |  |  |  |  |
|  |  | CNM-1927-20^T^ ([GCA_052527415.1](https://www.ncbi.nlm.nih.gov/datasets/genome/GCA_052527415.1/)) | 12.8 | | | [10.1 - 16.1] | | | | 19.5 | | | | [17.3 - 21.9] | | | | 13.2 | | | | [10.8 - 16] | | | | | 13.20 | | | | | |  |  |  |

| **(E)** | **Species**  (ascession number) | **16S gene similarity to CNM-4043-24ᵀ** | **ANI with**  **CNM-4043-24ᵀ** | **16S gene similarity to CNM-1927-20ᵀ** | **ANI with**  **CNM-1927-20ᵀ** |
| --- | --- | --- | --- | --- | --- |
|  | *L. taurinensis*  (NR_043927.1) | 95.24 | 70.24 | 95.38 | 68.64 |
|  | *L. rubrilucens*  (NR_036991.1) | 95.18 | 70.24 | 94.96 | – |
|  | *L. rowbothamii*  (NR_036804.1) | 95.01 | 68.29 | 94.08 | – |
|  | *L. maceachernii*  (NR_041790.1) | – | – | 95.65 | 70.20 |
|  | *L. micdadei*  (NR_041791.1) | – | – | 95.43 | 68.89 |
|  | *L. impletisoli*  (NR_041321.1) | – | – | 95.18 | 68.05 |
|  | *L. yabuuchiae*  (NR_041322.1) | – | – | 95.12 | 68.52 |
|  | *L. norrlandica*  (KJ796839.1) | – | – | 95.10 | 69.41 |
|  | *L. londiniensis*  (NR_044963.1) | – | – | 95.05 | 68.26 |
|  | *L. nagasakiensis*  (NR_116420.1) | – | – | 95.01 | 69.20 |
|  | *L. spiritensis*  (NR_117699.1) | – | – | 95.00 | 68.97 |

**Supplementary Table S4.** Enzymatic activity. Main biochemical features assayed using the API Zym and API 20 NE miniature systems for *L. pelazae* (CNM-1927-20^T^, CECT 31282) and *L. navarrensis* (CNM-4043-24^T^, CECT 31283) along with the results reported in the literature for the related strains *L. beliardensis* (Lo Presti et al. 2001), *L. gresilensis* (Lo Presti et al. 2001), *L. busanensis* (Park et al. 2003); *L. birminghamensis* (Wilkinson et al. 1987) and *L. quinlivanii* (Benson et al. 1989). Abbreviations: +, positive; -, negative activity; w: weak; S: strong. NR, not reported.

| **API ZYM** enzymes | **CNM-1927-20^T^**  **(CECT 31282)** | | | **CNM-4043-24^T^**  **(CECT 31283)** | | | *L.*  *beliardensis* (Montbéliard A1^T^) | | *L.*  *gresilensis* (Gréoux 11 D13^T^) | | | | *L.*  *busanensis* (K9951^T^) | | *L.*  *birminghamensis* (1407-AL-H) | | | *L.*  *quinlivanii* (CCUG 31234 A) | | | |  |
| --- | --- | --- | --- | --- | --- | --- | --- | --- | --- | --- | --- | --- | --- | --- | --- | --- | --- | --- | --- | --- | --- | --- |
| Alkaline phosphatase | + | | s | | | + | | | | + | | + | | | | NR | | | + | |  |  |
| Esterase (C4) | + | | w | | | + | | | | + | | + | | | | NR | | | - | |  |  |
| Esterase lipase (C8) | w | | - | | | + | | | | + | | + | | | | NR | | | + | |  |  |
| Lipase (C14) | - | | - | | | - | | | | - | | - | | | | NR | | | - | |  |  |
| Leucine arylamidase | + | | w | | | + | | | | + | | + | | | | NR | | | + | |  |  |
| Valine arylamidase | w | | w | | | + | | | | + | | + | | | | NR | | | + | |  |  |
| Cystine arylamidase | w | | - | | | - | | | | + | | - | | | | NR | | | + | |  |  |
| Trypsin | - | | - | | | - | | | | - | | - | | | | NR | | | - | |  |  |
| α-Chymotrypsin | - | | - | | | - | | | | - | | - | | | | NR | | | - | |  |  |
| Acid phosphatase | s | | w | | | + | | | | + | | + | | | | NR | | | + | |  |  |
| Naphthol-AS-BI-phosphohydrolase | + | | w | | | + | | | | + | | + | | | | NR | | | + | |  |  |
| α-Galactosidase | - | | - | | | - | | | | - | | - | | | | NR | | | - | |  |  |
| β-Galactosidase | - | | - | | | - | | | | - | | - | | | | NR | | | - | |  |  |
| β-Glucuronidase | - | | - | | | - | | | | - | | - | | | | NR | | | - | |  |  |
| α-Glucosidase | - | | - | | | - | | | | - | | - | | | | NR | | | - | |  |  |
| β-Glucosidase | - | | - | | | - | | | | - | | - | | | | NR | | | - | |  |  |
| N-Acetyl- | - | | - | | | - | | | | - | | - | | | | NR | | | - | |  |  |
| β-glucosaminase |  | |  | | |  | | | |  | |  | | | |  | | |  | |  |  |
| α-Mannosidase | - | | - | | | - | | | | - | | - | | | | NR | | | - | |  |  |
| β-Fucosidase | - | | - | | | - | | | | - | | - | | | | NR | | | - | |  |  |
| **API 20 NE** Enzymes/reactions | | **CNM-1927-20^T^**  **(CECT 31282)** | | | **CNM-4043-24^T^**  **(CECT 31283)** | | | *L.*  *beliardensis* (Montbéliard A1^T^) | | | *L.*  *gresilensis* (Gréoux 11 D13^T^) | | | *L.*  *busanensis* (K9951^T^) | | | *L.*  *birminghamensis* (1407-AL-H) | | | *L.*  *quinlivanii* (CCUG 31234 A) | | |
| Reduction nitrates-nitrites | | - | | | - | | | - | | | - | | | - | | | NR | | | NR | | |
| Reduction nitrates-nitrogen | | - | | | - | | | - | | | - | | | - | | | NR | | | NR | | |
| Indol production | | - | | | - | | | - | | | - | | | - | | | - | | | NR | | |
| Fermentation (glucose) | | - | | | - | | | - | | | - | | | NR | | | NR | | | NR | | |
| Arginine dihidrolase | | - | | | - | | | NR | | | NR | | | NR | | | NR | | | NR | | |
| Urease | | - | | | - | | | - | | | - | | | - | | | NR | | | NR | | |
| Hydrolisis (β-glucosidase) | | - | | | - | | | NR | | | NR | | | NR | | | NR | | | NR | | |
| Hydrolisis (protease) | | - | | | - | | | + | | | + | | | + | | | NR | | | NR | | |
| Β-galactosidase | | - | | | - | | | NR | | | NR | | | NR | | | NR | | | NR | | |
| Assimilation: | |  | | |  | | |  | | |  | | |  | | |  | | |  | | |
| Glucose | | - | | | - | | | NR | | | NR | | | NR | | | NR | | | NR | | |
| Arabinose | | - | | | - | | | NR | | | NR | | | NR | | | NR | | | NR | | |
| Mannose  N-acetyl- | | - | | | - | | | NR | | | NR | | | NR | | | NR | | | NR | | |
| glucosamine | | - | | | - | | | NR | | | NR | | | NR | | | NR | | | NR | | |
| Maltose | | - | | | - | | | NR | | | NR | | | NR | | | NR | | | NR | | |
| Potassium gluconate | | - | | | - | | | NR | | | NR | | | NR | | | NR | | | NR | | |
| Capric acid | | - | | | - | | | NR | | | NR | | | NR | | | NR | | | NR | | |
| Adipic acid | | - | | | - | | | NR | | | NR | | | NR | | | NR | | | NR | | |
| Maleate | | - | | | - | | | NR | | | NR | | | NR | | | NR | | | NR | | |
| Trisodium | | - | | | - | | | NR | | | NR | | | NR | | | NR | | | NR | | |
| citrate | | - | | | - | | | NR | | | NR | | | NR | | | NR | | | NR | | |
| Phenylacetic acid | | - | | | - | | | NR | | | NR | | | NR | | | NR | | | NR | | |
| Cytochrome oxidase | | - | | | - | | | NR | | | NR | | | NR | | | NR | | | NR | | |

**Supplementary Table S5.** Antibiotic susceptibility testing for *L. pelazae* (CNM-1927-20^T^) and *L. navarrensis* (CNM-4043-24^T^). MIC values determined by the Broth microdilution method (BMD) for CNM-1927-20^T^ for azithromycin, doxycycline, lefamulin, levofloxacin and rifampicin, and MIC values determined by gradient methods (ETEST**^®^**) for both strains for azithromycin, doxycycline, levofloxacin and rifampicin. All values are expressed in µg/mL. *Interpretation of MIC values according to EUCAST Guidance Document [1]. Abbreviations: NR: not reported.

| **BMD** | | | | | |
| --- | --- | --- | --- | --- | --- |
| **Antimicrobial agent**  (range) | CNM-1927-20^T^ | | | | **ECOFFs***  (Not standarised) |
|  | Mean ± SD | Median | N | |  |
| Azitromycin (0.08-4) | 8 ± 0.00 | 8 | | 3 | 0.125 |
| Doxycycline (0.125-64) | 3 ± 1.7 | 4 | | 3 | 2 |
| Lefamulin (0.08-4) | 0.04 ± 0.02 | 0.03 | | 3 | NR |
| Levofloxacin (0.0025-0.125) | 0.14 ± 0.1 | 0.25 | | 3 | 0.125 |
| Rifampicin (0.00025-0.125) | 0.003 ±0.01 | 0.078 | | 3 | 0.008 |

| **ETEST^®^** | | | | | | | |
| --- | --- | --- | --- | --- | --- | --- | --- |
| **Antimicrobial agent**  (range) | CNM-1927-20^T^ | | | CNM-4043-24^T^ | | | **ECOFFs*** |
|  | Mean ± SD | Median | N | Mean ± SD | Median | N | (Not standarised) |
| Azitromycin (0.016-256) | 1.75 ± 0.29 | 1.75 | 4 | 0.16 ± 0.03 | 0.19 | 5 | 0.25 |
| Doxycycline (0.016-256) | 1.56 ± 0.59 | 1.75 | 4 | 1.69 ± 0.24 | 1.62 | 4 | 8 |
| Levofloxacin (0.002-32) | 0.28 ± 0.06 | 0.25 | 5 | 0.12 ± 0.00 | 0.12 | 5 | 0.25 |
| Rifampicin (0.02-32) | 0.03 ± 0.01 | 0.32 | 5 | 0.32 ± 0.41 | 0.12 | 4 | 0.032 |

[1]. European Committee on Antimicrobial Susceptibility Testing (EUCAST). *Guidance document on Antimicrobial Susceptibility Testing of Legionella pneumophila*. EUCAST; 2021. Available from: https://www.eucast.org/news-detail/legionella-ast-guidance-updated/

## 1.2 Supplementary Figures

**Supplementary Figure S1** Maximum-likelihood phylogenetic tree based on 16S rRNA gene (A) and *mip* gene (B) core genome alignment]. The tree was inferred using IQ-TREE under the best-fit substitution model selected according to the Bayesian Information Criterion. Branch support was assessed using 1,000 ultrafast bootstrap (UFBoot) replicates and 1,000 SH-aLRT replicates. Only support values ≥70% (UFBoot) and ≥80% (SH-aLRT) are shown at the nodes. SH-aLRT and UFBoot values are shown as SH-aLRT/UFBoot. GenBank accession numbers are indicated in parentheses. Novel species proposed in this study are highlighted in bold. Scale bar represents the number of substitutions per site.

**Supplementary Figure S2** Maximum-likelihood phylogenomic tree showing the relationships of CNM-1927-20ᵀ and CNM-4043-24ᵀ with 54 representative *Legionella* genomes. The tree was inferred using the chewBBACA cgMLST approach, as described in the Materials and Methods section. Bootstrap values, calculated from 1,000 replicates, are indicated next to the branches. Branch lengths represent the number of substitutions per site, indicating evolutionary divergence among taxa. GenBank accession numbers are provided in parentheses. Novel species described in this study are highlighted in bold.

**Supplementary Figure S3** Distribution of the ten most abundant COG functional categories in strains CNM-1927-20ᵀ (purple bars; *Legionella pelazae* sp. nov.) and CNM-4043-24ᵀ (green bars; *Legionella navarrensis* sp. nov.). Values are expressed as percentages of total COG-annotated proteins.

**(A)**

**(B)**

**Supplementary Figure S4** (A) Comparative heatmap of virulence-associated homologues detected in strains CNM-1927-20ᵀ and CNM-4043-24ᵀ and closely related *Legionella* species. Presence/absence was inferred using ABRicate (version 1.4.0) with relaxed thresholds (≥60% identity and ≥80% coverage) against the VFDB database.

(B) Comparative heatmap of antimicrobial resistance-associated homologues detected in strains CNM-1927-20ᵀ and CNM-4043-24ᵀ and related *Legionella* species. Screening was performed using ABRicate (version 1.4.0) against the NCBI AMRFinderPlus v3.12.8 database with relaxed thresholds (≥60% identity and ≥80% coverage).

Yellow indicates presence, and purple indicates absence of homologous genes.
